# Activating transcription factor-2 supports the antioxidant capacity and ability of human mesenchymal stem cells to prevent asthmatic airway inflammation

**DOI:** 10.1038/s12276-023-00943-z

**Published:** 2023-02-10

**Authors:** Hyein Ju, HongDuck Yun, YongHwan Kim, Yun Ji Nam, Seungun Lee, Jinwon Lee, Seon Min Jeong, Jinbeom Heo, Hyungu Kwon, You Sook Cho, Gowun Jeong, Chae-Min Ryu, Dong-Myung Shin

**Affiliations:** 1 Department of Biomedical Sciences, Asan Medical Center, University of Ulsan College of Medicine, Seoul, 05505 South Korea; 2 Department of Physiology, University of Ulsan College of Medicine, Seoul, 05505 South Korea; 3 Division of Allergy and Clinical Immunology, Department of Internal Medicine, Asan Medical Center, University of Ulsan College of Medicine, Seoul, 05505 South Korea; 4 AI Recommendation, T3K, SK Telecom, Seoul, 04539 South Korea; 5 Center for Cell Therapy, Asan Medical Center, Seoul, 05505 South Korea

**Keywords:** Mesenchymal stem cells, Asthma

## Abstract

Glutathione (GSH), an abundant nonprotein thiol antioxidant, participates in several biological processes and determines the functionality of stem cells. A detailed understanding of the molecular network mediating GSH dynamics is still lacking. Here, we show that activating transcription factor-2 (ATF2), a cAMP-response element binding protein (CREB), plays a crucial role in maintaining the level and activity of GSH in human mesenchymal stem cells (MSCs) by crosstalking with nuclear factor erythroid-2 like-2 (NRF2), a well-known master regulator of cellular redox homeostasis. Priming with ascorbic acid 2-glucoside (AA2G), a stable vitamin C derivative, increased the expression and activity of ATF2 in MSCs derived from human embryonic stem cells and umbilical cord. Subsequently, activated ATF2 crosstalked with the CREB1-NRF2 pathway to preserve the GSH dynamics of MSCs through the induction of genes involved in GSH synthesis (*GCLC* and *GCLM*) and redox cycling (*GSR* and *PRDX1*). Accordingly, shRNA-mediated silencing of *ATF2* significantly impaired the self-renewal, migratory, proangiogenic, and anti-inflammatory capacities of MSCs, and these defects were rescued by supplementation of the cells with GSH. In addition, silencing *ATF2* attenuated the ability of MSCs to alleviate airway inflammatory responses in an ovalbumin-induced mouse model of allergic asthma. Consistently, activation of ATF2 by overexpression or the AA2G-based priming procedure enhanced the core functions of MSCs, improving the in vivo therapeutic efficacy of MSCs for treating asthma. Collectively, our findings suggest that ATF2 is a novel modulator of GSH dynamics that determines the core functionality and therapeutic potency of MSCs used to treat allergic asthma.

## Asthma: Antioxidant-boosting protein improves stem cell treatment

A cellular protein that promotes a key antioxidant will be a crucial component in stem cell therapies for allergic asthma. Stem cells derived from umbilical cords have been proposed as treatments for incurable allergic asthma, due to their ability to combat inflammation and regenerate damaged cells. Now, Dong-Myung Shin at University of Ulsan College of Medicine in Seoul, South Korea, and co-workers have shown that the activating transcription factor 2 (ATF2) acts to maintain healthy levels of the antioxidant glutathione, which is essential for the effectiveness of stem cell therapy. Specifically, ATF2 interplays with a specific nuclear protein to activate genes involved in glutathione synthesis. The researchers showed that the ability of MSC treatments to reduce airway inflammation in asthmatic mouse models was greatly reduced by silencing ATF2, and enhanced by its over-expression.

## Introduction

Asthma is the most common chronic disease of the lungs in children and adults. The prevalence of asthma has doubled in the past decade, leading to a substantial global health and economic burden. Asthma is an allergic disease that is characterized by a combination of inflammation and structural remodeling in the airways^1^. The resulting airway obstruction causes breathing difficulties, wheezing, shortness of breath, and coughing. Immune responses mediated by innate lymphoid cells and T helper 2 (Th2) cells contribute to allergic airway inflammation and fibrosis, causing permanent deterioration in pulmonary function^2–4^. Asthma patients are grouped into one of four or five categories and are treated in a stepwise manner, depending on symptom severity or extent of disease. Inhaled corticosteroids, long-acting β2-adrenergic receptor agonists, long‑acting muscarinic antagonists, and leukotriene receptor antagonists are used as asthma-control drugs, and an IgE-specific monoclonal antibody is used to treat the most severe form of the disease^5^. Although this stepwise approach has improved the management of asthma and reduced dependency on inhaled short-acting bronchodilators for symptom relief, none of the currently available treatments can alter the progression of the disease; hence, there is an urgent need to develop novel therapies.

Preclinical and clinical studies have suggested beneficial effects of mesenchymal stem cells (MSCs) in treating incurable allergic asthma^6–10^. These progenitor cells are typically derived from adult tissues, such as the bone marrow, adipose tissue, and umbilical cord (UC) or UC blood, and can also be established by differentiation from pluripotent stem cells (PSCs), including embryonic stem cells (ESCs) and induced PSCs (iPSCs)^11–14^. The therapeutic effects of MSCs are thought to be attributable to their multipotency and ability to directly regenerate damaged cells in target tissues. In addition, MSCs can have indirect effects by providing growth factors, mediating cell‒cell interactions, and supplying matrix proteins to modulate the microenvironment of damaged target tissues and facilitate regeneration^15,16^. In particular, the anti-inflammatory and immunomodulatory functions of MSCs are achieved by inhibiting the activation, proliferation, and function of immune cells, including T cells, B cells, innate lymphoid cells, natural killer cells, and antigen-presenting cells^17,18^.

Despite the multifactorial benefits of MSCs, their clinical application has been hindered by limited therapeutic efficacy and a lack of knowledge of their precise mode of action. The high therapeutic potency of primitive MSCs is reportedly lost after large-scale ex vivo expansion, which is required to obtain a sufficient number for therapeutic purposes, due to an accumulation of epigenetic abnormalities and oxidative stress provoked by supraphysiological stimulations^19^. We have described several ex vivo expansion methods to preserve the primitiveness of MSCs, including i) enriching and preserving small-sized cells^20^, ii) enhancing the antioxidant capacity by real-time monitoring of glutathione (GSH) dynamics^9,21^, and iii) enhancing cell migration and engraftment activity by priming with small molecules^22^. In addition, we recently reported that supplementation with small compounds without genetic manipulation enables the enrichment and expansion of small primitive MSCs with a high antioxidant and engraftment capacity, which was termed the **P**rimed/**F**resh/**O**CT4 (PFO) enrichment procedure^23^. All of these procedures enhance the levels and dynamics of GSH, which is essential to maintain the stemness and therapeutic efficacy of human MSCs^9,21,23^.

Mechanistically, MSCs with high GSH dynamics display activation of the cyclic adenosine monophosphate (cAMP)-response element (CRE) binding protein-1 (CREB1) and nuclear factor erythroid-2 like-2 (NRF2) pathway, leading to the induction of genes involved in GSH synthesis (*GCLC* and *GCLM*) and redox cycling (*GSR* and *PRDX1*)^10,21,23^. The intracellular levels and dynamics of GSH in MSCs can be improved by pretreatment/priming with forskolin (FSK), a CREB1 activator, or by priming with ascorbic acid 2-glucoside (AA2G), a stable vitamin-C (VitC) derivative that activates CREB1 and in turn upregulates NRF2 target genes responsible for GSH synthesis and redox cycling^10,21^. The biological effects of these GSH-enhancing conditions stimulate the core functions of MSCs derived from various sources, including human ESCs and adult tissues such as the UC and bone marrow. Notably, in previous studies, the in vivo therapeutic effects of MSCs were enhanced by improving GSH dynamics in an experimental asthma animal model and a humanized graft-versus-host disease mouse model^10,23^.

In this study, we demonstrate that activating transcription factor-2 (ATF2), a member of the leucine zipper domain-containing CREB/ATF transcription factor family, plays a key role in modulating GSH dynamics and determining the core functionality and therapeutic potency of MSCs used to treat allergic asthma.

## Materials and methods

### Study approval

Human UC samples were obtained from healthy full-term newborns after obtaining written informed consent. All procedures were performed in accordance with the guidelines of the Ethics Committee on the Use of Human Subjects at Asan Medical Center (IRB#: 2015-0303). All animal experiments were approved by and performed in accordance with the guidelines and regulations of the Institutional Animal Care and Use Committee of the University of Ulsan College of Medicine (IACUC-2019-12-221 and IACUC-2019-12-325).

### Culture of MSCs

Human ESC-derived MSCs (hES-MSCs) were established by differentiation from H9 hESCs^11,12^ and were maintained in EGM2-MV medium (Lonza, San Diego, CA, USA) on plates coated with rat tail collagen type I (Sigma-Aldrich, St. Louis, MO, USA), as described previously^9,10,13^. Human UC MSCs (hUC-MSCs) were isolated from UCs, as described previously^24^, and were grown in low-glucose DMEM containing 10% heat-inactivated fetal bovine serum (HyClone, Pittsburgh, PA, USA), 5 ng/mL human epidermal growth factor (Sigma-Aldrich), 10 ng/mL basic fibroblast growth factor, and 50 ng/mL long-R3 insulin-like growth factor-1 (ProSpec, Rehovot, Israel), as described previously^10,21,22^. All MSCs used in this study were expanded for fewer than seven passages to ensure their functionality and were maintained at 37 °C in a humidified atmosphere containing 5% CO_2_.

For GSH-enhancing priming, MSCs were plated at a density of 7 × 10^4^ cells/mL in culture medium with the indicated concentration of AA2G (Sigma-Aldrich) for 3 days or 2 µM FSK (Sigma-Aldrich) for the indicated number of hours. The intracellular GSH level was rescued by supplementation with 0.125 µM GSH ethyl ester (GSH-EE; Sigma-Aldrich) for 4 h. The PFO procedure was performed by supplementation with AA2G, followed by treatment with low concentrations of sphingosine-1-phosphate (S1P) and valproic acid (VPA), as previously described^23^. In brief, MSCs were plated at a density of 7 × 10^4^ cells/mL and maintained in culture medium with 0.74 mM (Sigma-Aldrich) for two days. One day before the functional evaluation, 50 nM S1P and 0.5 mM VPA (Sigma-Aldrich) were added to the culture medium containing 0.74 mM AA2G.

### RNA interference and ectopic expression of *ATF2*

For knockdown (KD) of *ATF2*, three independent shRNAs targeting human *ATF2* were cloned into the pLenti6/Block-iT lentiviral vector (Invitrogen/Thermo Fisher Scientific, Waltham, MA, USA). For ectopic expression, the open reading frame (ORF) of human *ATF2* in the pDONR223 plasmid (Addgene plasmid # 82889) was cloned into the pEZ-Lv235 (#EZ016, GeneCopoeia, Rockville, MD, USA) plasmid using the Gateway Technology reaction in accordance with the manufacturer’s instructions (Invitrogen/Thermo Fisher Scientific). Lentiviruses carrying each *ATF2* shRNA or human *ATF2* ORF were produced and used to infect hES-MSCs or hUC-MSCs, as described previously^25^. The sequences of the shRNAs are shown in Supplementary Table 1. The ORF of human *ATF2* was kindly provided to us by Jesse Boehm, Matthew Meyerson, and David Root.

### In vitro cell proliferation, self-renewal, multipotency, and migration of MSCs

Several in vitro assays were performed to assess the cellular activities of MSCs. An MTT assay (Sigma-Aldrich) was used to assess cell proliferation, and a colony forming unit-fibroblast (CFU-F) assay was used to assess self-renewal. Multipotency (in vitro differentiation into chondrogenic, osteogenic, or adipogenic lineages) and transwell migration in response to platelet-derived growth factor (PDGF; 10 ng/mL PDGF-AA, R&D Systems, Minneapolis, MN, USA) were also assessed. Angiogenesis was quantified using Matrigel, and in vitro anti-inflammation was analyzed as described previously^8–10,20,21^. The digital images generated in these assays were assessed quantitatively using Image-Pro 5.0 software (Media Cybernetics, Rockville, MD, USA).

### Real-time monitoring of the GSH-recovery capacity of living MSCs

Real-time monitoring of the GSH-recovering capacity (GRC) of every living cell under different culture conditions was achieved using an Operetta High-Content Imaging System (HH12000000; PerkinElmer, Waltham, MA, USA) at ×200 or ×400 magnification, as described previously^21^. This system provides a nondestructive, integrated, and image-based high-throughput method for analyzing the qualitative and quantitative aspects of GSH dynamics in living MSCs. The GRC assay was based on the unique properties of FreSHtracer (Fluorescent real-time thiol tracer; Cell2in, Inc., Seoul, Korea), a reversible chemical probe for GSH^9,26^. Upon reacting with GSH, FreSHtracer shows a spectral shift in the λ_max_ of its ultraviolet‒visible absorption from 520 nm to 430 nm, resulting in decreased fluorescence emission intensity at 580 nm (F_580_, λ_ex_ 520 nm) and increased fluorescence intensity at 510 nm (F_510_, λ_ex_ 430 nm)^9,26^. Thus, to determine the fluorescence ratios of FreSHtracer, fluorescence emissions were measured at 510 and 580 nm after excitation at 430 and 520 nm, respectively. These fluorescence signals were analyzed using Harmony High-Content Imaging and Analysis Software 3.1 (PerkinElmer) in confocal mode. The GSH dynamics indices, related initial fluorescence ratios (representing baseline total GSH), and slopes after diamide treatment (representing the GRC) of each plot are presented in Supplementary Dataset 1.

### Asthma animal model

Asthma was induced in 6-week-old female BALB/c mice (JA Bio, Suwon, Korea) by sensitization with intraperitoneal injections of 100 µg of ovalbumin (OVA, Sigma-Aldrich) and 2 mg of aluminum hydroxide (Sigma-Aldrich) on Days 0 and 7, followed by allergen challenge via intranasal injection of 50 μg of OVA on Days 14, 15, 16, 21, 22, and 23, as reported previously^27^. After 17 days, 3 × 10^5^ hUC-MSCs stably expressing a control (shCTR) or *ATF2*-specific (sh*ATF2*) shRNA construct were suspended in 100 µL of phosphate-buffered saline (PBS) and injected via the tail vein. The same procedure was applied for the administration of hUC-MSCs or hES-MSCs, which were expanded under normal (naïve) culture conditions or using the PFO procedure. PBS alone was injected as a control (sham and asthma groups). Mice were randomly allocated to treatment groups, and the order of allergen sensitization or challenge and injection of MSCs or vehicle was randomized. Treatment groups were masked to investigators who participated in the therapeutic evaluation procedures.

### Analysis of airway inflammation

For mechanistic insights into MSC therapy, airway inflammation was evaluated by histological examination and bronchoalveolar lavage fluid (BALF) analysis, as well as via analyses of the expression levels of cytokine genes and proteins in the lung, as reported previously^9,10^. Therapeutic outcomes were analyzed using two independent sets of five animals per group. All histological, BALF, and cytokine analyses were performed by blinded investigators.

For analysis of engraftment of the hUC-MSCs, human β2-microglobulin (hB2M) was detected using a mouse monoclonal antibody (SC80668; Santa Cruz Biotechnology, Santa Cruz, CA, USA) and an Alexa Fluor 488-labeled anti-mouse secondary antibody (Invitrogen).

Differentiation lineage was determined by costaining for hB2M and prosurfactant protein C (SFTPC) rabbit polyclonal antibody (ab90716; Abcam, Cambridge, UK) and visualization with an Alexa Fluor 546-labeled anti-rabbit secondary antibody (Invitrogen). Nuclei were counterstained using DAPI (Sigma-Aldrich). Digital images were selected at random from each slide and used for quantification using Image-Pro 5.0 software.

For gene expression analyses, total RNA was isolated from frozen lung tissues using the RNeasy Mini Kit (Qiagen, Hilden, Germany) and treated with DNase I (Qiagen). Total RNA (800 ng) was reverse-transcribed with TaqMan Reverse Transcription Reagent (Applied Biosystems, Foster City, CA), and the threshold cycle (Ct) was subsequently determined via real-time quantitative PCR (RQ-PCR), as described previously^28^. The relative expression level of each target gene was determined using the 2^-ΔΔCt^ method, with *Gapdh* as the endogenous control gene. All primers used in the RQ-PCR assay are listed in Supplementary Table 2.

### Statistical analysis

Statistical significance was evaluated by the nonparametric Mann‒Whitney test and one-way or two-way ANOVA with the Bonferroni post hoc test using GraphPad Prism 7.0 software (GraphPad Software, La Jolla, CA, USA); *p* < 0.05 was considered statistically significant.

## Results

### Priming to enhance GSH levels activates ATF2 in MSCs

**Fig. 1:**
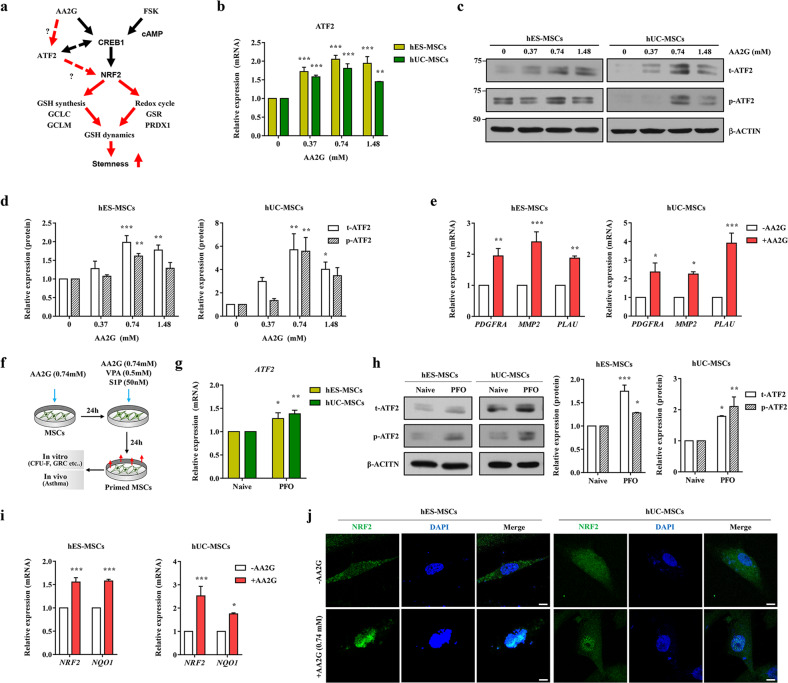
AA2G priming activates ATF2 and NRF2 in human MSCs. **a** A schematic overview of the ATF2 and CREB1-NRF2 cascades involved in GSH dynamics in human MSCs. **b** RQ-PCR analysis (*n* = 4) of the *ATF2* transcript in the AA2G-treated hES-MSCs and hUC-MSCs. **c** Western blot analyses (*n* = 3) of total (t-ATF2) and phosphorylated ATF2 (p-ATF2) proteins in the AA2G-treated hES-MSCs and hUC-MSCs. The expression level of β-actin was used as a loading control. Molecular weight marker sizes (kD) are shown on the left. **d** Quantification of the western blotting data described in **c**. **e** RQ-PCR analyses (*n* = 4) of ATF2 target genes following treatment of hES-MSCs and hUC-MSCs with AA2G for 72 h. **f** Schematic summary of the PFO procedure, which included supplementation with 0.74 mM AA2G for two days, followed by further stimulation with 50 nM sphingosine 1-phosphate (S1P) and 0.5 mM valproic acid (VPA) one day before functional evaluation. RQ-PCR (*n* = 4; **g**) and western blot analyses (*n* = 3; **h**) for the expression of ATF2 in hES-MSCs and hUC-MSCs under normal (naïve) or PFO culture conditions. **i** RQ-PCR analyses (*n* = 4) of *NRF2* and *NQO1* in the hES-MSCs and hUC-MSCs primed with 0.74 mM AA2G for 72 h. **j** Representative confocal microscopy images of the NRF2 protein (green) in the hES-MSCs and hUC-MSCs treated with or without AA2G. Magnification, ×1000. Scale bar, 10 µm. Nuclei were stained with DAPI (blue). **b**, **d–i** Data are represented as ratios relative to the nontreated cells (-AA2G). All quantification results are shown as the mean ± SEM (*p < 0.05, **p < 0.01, ***p < 0.001 compared with nontreated cells, via two-way ANOVA).

In a previous transcriptome analysis, a population of hES-MSCs with a high level of GSH (GSH^High^) was characterized by upregulation of the genes encoding ATF2 and other structurally related activating protein-1 (AP1) proteins, such as JUN, JUNB, and FRA1^21,29^. This finding was validated by increased levels of the proteins encoded by these genes, as well as their phosphorylated active counterparts, in GSH^High^ hES-MSCs^21^. ATF2 forms a heterodimer with several AP1 proteins, binds to the CRE to regulate gene expression and is activated by several extracellular stimuli, such as hypoxia, oxidative stress, and DNA damage^29–31^. Therefore, we examined whether priming to enhance GSH levels could activate cAMP-dependent ATF2 and affect the CREB1-NRF2 signaling cascade in MSCs derived from different sources (Fig. 1a). To this end, we examined the expression level and activity of ATF2 in hES-MSCs and hUC-MSCs cultured in medium with different concentrations (0, 0.37, 0.74, and 1.48 mM) of AA2G for 72 h. RQ-PCR and western blot analyses showed that the transcript and protein levels of ATF2 were increased by AA2G priming in both hES-MSCs and hUC-MSCs (Fig. 1b–d), peaking at the 0.74 mM AA2G concentration. This upregulation was accompanied by an increase in the level of active ATF2 protein, which is phosphorylated at threonine 69 (Thr69) and 71 (Thr71) via mitogen-activated protein kinases such as p38, JNK, and ERK^32^. Consistent with these results, the expression levels of a subset of ATF2 target genes, including *PDGFRA*, *MMP2*, and *PLAU*, were increased following AA2G priming, and this effect was greater in hUC-MSCs than in hES-MSCs (Fig. 1e and Supplementary Fig. 1a).

We previously reported that further stimulation of AA2G-primed MSCs with a low concentration of S1P and VPA was beneficial for preserving primitive MSCs, characterized morphologically by their small size and high GSH dynamics^23^. In this regard, we examined whether the expression of ATF2 could be affected by the PFO procedure based on the combination of three small molecules, AA2G, S1P, and VPA (Fig. 1f). The PFO procedure increased ATF2 transcript and protein expression levels in both hES- and hUC-MSCs (Fig. 1g, h), resulting in the upregulation of ATF2 target genes (Supplementary Fig. 1b).

Next, we examined the effect of FSK priming on ATF2 expression. FSK treatment of hUC-MSCs increased the *ATF2* transcript level only minimally (Supplementary Fig. 1c) but significantly increased the levels of total and phosphorylated ATF2 proteins, with peaks occurring 4 and 2 h after FSK priming, respectively (Supplementary Fig. 1d). In hES-MSCs, FSK priming had slight effects on the levels of the ATF2 transcript and protein (Supplementary Fig. 1e, f). Overall, these data demonstrate that GSH-enhancing priming conditions can regulate the expression and activity of ATF2 in human MSCs, depending on the particular cell context and priming factors used. Since AA2G treatment stably activated ATF2 in both hES-MSCs and hUC-MSCs, we used the optimal dose of 0.74 mM AA2G in subsequent studies.

### ATF2 regulates redox homeostasis in MSCs by crosstalking with the NRF2 pathway

**Fig. 2:**
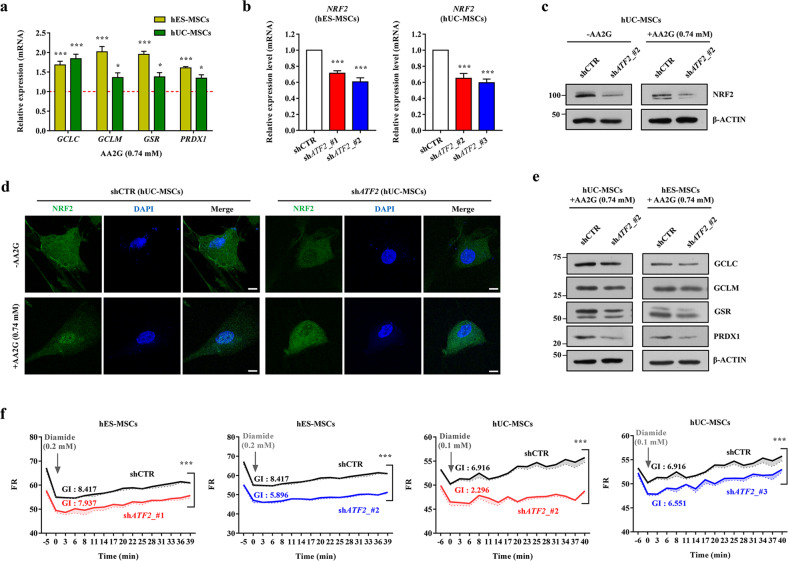
Crosstalk between the ATF2 and NRF2 pathways in AA2G-primed MSCs. **a** RQ-PCR analyses (*n* = 4) of CREB1-NRF2-dependent GSH synthesis (*GCLC* and *GCLM*) and redox cycling (*GSR* and *PRDX1*) genes in AA2G-primed MSCs. Expression levels were calculated as the ratio of the value of AA2G-primed MSCs to the nontreated cells (set to 1; see the red dotted line). RQ-PCR analysis (*n* = 4) of the *NRF2* transcript (**b**) and western blot analyses of NRF2 protein (**c**) in the MSCs expressing a scrambled control shRNA (shCTR) or an *ATF2*-specific shRNA (sh*ATF2*). Two independent sh*ATF2* constructs were used. **d** Representative confocal microscopy images of the NRF2 protein (green) in the hUC-MSCs treated with or without AA2G and expressing shCTR or sh*ATF2*. Nuclei were stained with DAPI (blue). Magnification, ×1000. Scale bar, 10 µm. **e** Western blot analyses of NRF2 target genes in the AA2G-primed hES-MSCs and hUC-MSCs expressing shCTR or sh*ATF2*. Molecular weight marker sizes (kD) are shown on the left of the blots. **f** F_510_/F_580_ fluorescence ratio (FR) plots of the hES-MSCs and hUC-MSCs carrying the indicated shCTR or sh*ATF2* constructs. The GSH dynamics index (GI) for each sample (*n* = 3) was quantified based on both the initial FR (representing the baseline of total GSH) and the slope after 0.1 or 0.2 mM diamide treatment (representing the GRC). Representative images of F_510_ (GSH bound) and F_580_ (GSH free) fluorescence are shown in Supplementary Fig. 3b. All quantification results are shown as the mean ± SEM. Statistical analyses were performed via one-way (**b**) or two-way (**a** and **f**) ANOVA with Bonferroni *post hoc* tests (*p < 0.05, ***p < 0.001 compared with nontreated or shCTR cells).

We then investigated whether ATF2 can directly modulate the CREB1-NRF2 signaling cascade in MSCs. In both hES-MSCs and hUC-MSCs, AA2G priming increased the mRNA expression levels of *NRF2* and *NQO1*, a well-known NRF2 target gene (Fig. 1i), and stimulated translocation of the NRF2 protein into the nucleus, indicating activation of the NRF2 pathway (Fig. 1j). Consistent with these results, AA2G priming also increased the expression levels of genes related to GSH synthesis (*GCLC* and *GCLM*) and redox cycling (*GSR* and *PRDX1*) (Fig. 2a), which have been reported as targets of the CREB1-NRF2 pathway that play a role in the maintenance of redox homeostasis in MSCs^21^. NRF2 and its targets (*GCLC, GCLM*, *GSR*, and *PRDX1*) were decreased at the mRNA and protein levels in MSCs harboring shRNAs targeting ATF2 (sh*ATF2*) (Fig. 2b, c, and Supplementary Fig. 2a–d). Notably, KD of *ATF2* also impaired the AA2G-mediated nuclear translocation of the NRF2 protein (Fig. 2d and Supplementary Fig. 2e) and induction of the GSH-related genes targeted by CREB1-NRF2 (*GCLC, GCLM*, *PRDX1*, and *GSR*) (Fig. 2e and Supplementary Fig. 2f), suggesting an interplay between the ATF2 and CREB1-NRF2 signaling cascades in MSCs. The majority of ATF2 target genes activated by AA2G priming were downregulated by KD of *ATF2* (Supplementary Fig. 2g).

To investigate the biological relevance of these findings, we used a high-throughput GRC assay (Supplementary Fig. 3), which enables real-time monitoring of the qualitative and quantitative aspects of GSH dynamics in living cells using a reversible chemical probe^21^. Changes in the intracellular GSH level were monitored for approximately 1 h after exposure to diamide, a thiol-specific oxidant. KD of *ATF2* decreased the basal level of GSH and impaired the GRC following diamide treatment in both hES-MSCs and hUC-MSCs (Fig. 2f), indicating a crucial role of ATF2 in maintaining GSH dynamics in MSCs. Collectively, these results demonstrate that ATF2 acts as a novel mediator of redox homeostasis in MSCs by crosstalking with the NRF2 signaling cascade.

### ATF2 regulates the core functions of MSCs

To explore its role in maintaining the characteristics of MSCs that influence their therapeutic potency, we silenced *ATF2* in hES-MSCs by infecting the cells with lentiviruses harboring two independent sh*ATF2* constructs. KD of *ATF2* had little effect on the expression of surface markers characteristic of MSCs, including CD29, CD73, and CD105 (Supplementary Fig. 4a). Furthermore, KD of *ATF2* had little effect on the in vitro differentiation of hES-MSCs into the chondrogenic, adipogenic, and osteogenic lineages, which were evaluated by an increased level of cartilage proteoglycans (Alcian Blue staining), accumulation of lipid droplets (Oil Red O staining), and mineral deposition (Alizarin Red S staining), respectively (Supplementary Fig. 4b).

**Fig. 3:**
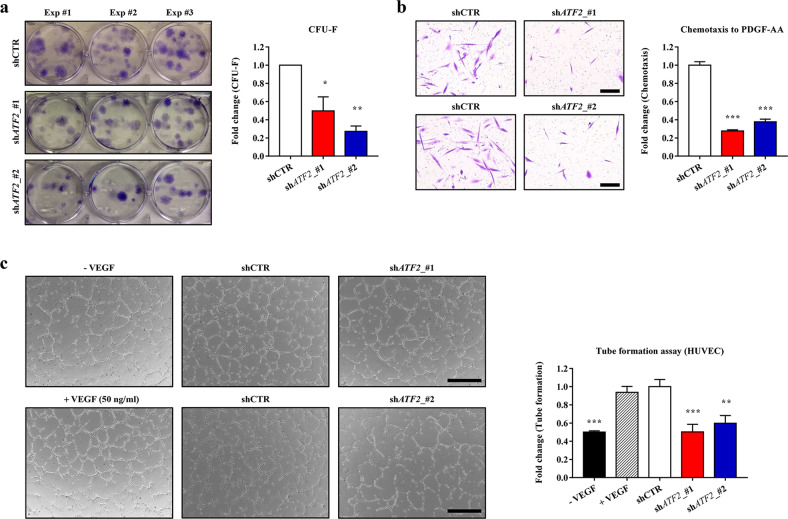
ATF2 is critical for maintaining the core functions of hES-MSCs. The effects of silencing *ATF2* in hES-MSCs on colony forming unit-fibroblast (CFU-F) potency (*n* = 3; **a**), chemotaxis (*n* = 7; **b**) in response to treatment with 10 ng/mL PDGF-AA, and angiogenesis in an in vitro Matrigel tube formation assay (*n* = 4; **c**). Cells expressed a scrambled control (shCTR) or *ATF2*-specific (sh*ATF2*) shRNA. Two independent sh*ATF2* constructs were used. Representative results for each assay are shown on the left (**b**: magnification, ×200; scale bar, 100 µm; **c**: magnification, ×40; scale bar, 200 µm). For the Matrigel tube formation assay, conditioned medium was prepared from the indicated hES-MSCs, and saline and recombinant human VEGF-A were used as negative and positive controls, respectively. Quantitative data are presented as ratios relative to the shCTR cells and are expressed as the mean ± SEM. Statistical analyses were performed via one-way ANOVA (*p < 0.05, **p < 0.01, ***p < 0.001 compared with shCTR cells).

KD of *ATF2* in hES-MSCs decreased the potency of CFU-F, which represents the presence of true clonogenic progenitor cells (Fig. 3a), but did not significantly affect cell proliferation (Supplementary Fig. 4c). A transwell chemotactic assay revealed that *ATF2*-KD hES-MSCs exhibited a severe defect in PDGF-stimulated cell migration (Fig. 3b). Furthermore, the ability of conditioned medium (CM) from *ATF2*-KD hES-MSCs to induce angiogenesis in a Matrigel tube formation assay was lower than that of CM from MSCs harboring a control shRNA (shCTR) construct (Fig. 3c).

**Fig. 4:**
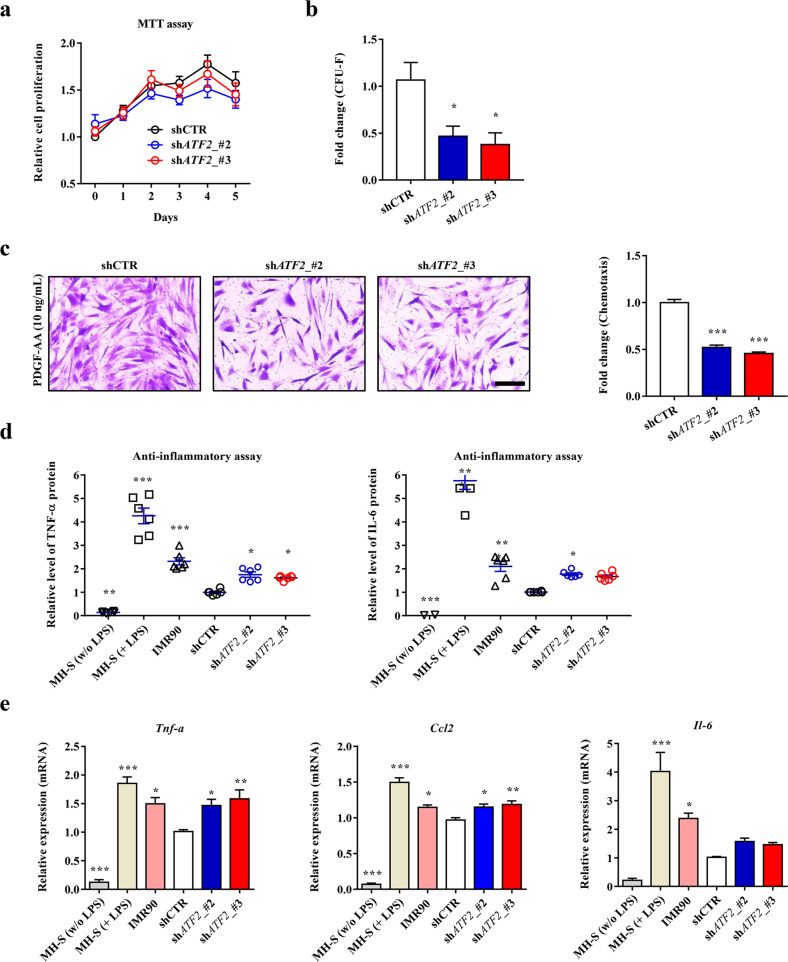
*ATF2*-silenced hUC-MSCs display defective therapeutic functions. Analyses of cell proliferation (*n* = 6; **a**), CFU-F potency (*n* = 5; **b**), and chemotaxis in response to treatment with 10 ng/mL PDGF-AA (*n* = 7; **c**) in hUC-MSCs harboring a scrambled (shCTR) or *ATF2*-specific (sh*ATF2*) shRNA. **c** Representative examples of chemotactic assays (magnification, ×200; scale bar, 100 µm) are shown next to the corresponding quantitative data. **d**, **e** Anti-inflammation assays using conditioned medium (CM) prepared from the AA2G-treated hUC-MSCs. **d** Quantification of TNF-α and IL-6 proteins secreted from MH-S cells that were stimulated with LPS for 8 h in the absence or presence of CM harvested from the indicated cells (*n* = 6). CM from IMR90 normal primary fibroblasts was used as a control. **e** RQ-PCR analyses of the expression levels of selected murine inflammatory genes in the LPS-stimulated MH-S cells (*n* = 6). All quantitative data are represented as fold changes relative to the shCTR group and are displayed as the mean ± SEM. Statistical analyses were performed via one-way ANOVA (*p < 0.05, **p < 0.01, ***p < 0.001 compared with shCTR cells).

As observed in hES-MSCs, KD of *ATF2* had minimal effects on the basic characteristics of MSCs, including surface marker phenotypes, multipotency, and cell proliferation (Fig. 4a, Supplementary Fig. 5a, b), but significantly impaired the core functions of hUC-MSCs, including the potency of CFU-F (self-renewal) and PDGF-responsive chemotaxis capacity (Fig. 4b, c, and Supplementary Fig. 5c).

To examine the anti-inflammatory response of MSCs, we collected CM from hUC-MSCs and applied it to MH-S murine alveolar macrophages that were pretreated with lipopolysaccharide (LPS). As reported previously^10,21,23^, CM from hUC-MSCs expressing shCTR significantly reduced secretion of the proinflammatory cytokines tumor necrosis factor-α (TNF-α) and interleukin-6 (IL-6) by LPS-stimulated MH-S cells, whereas CM collected from control cells (IMR90 human lung fibroblasts) did not (Fig. 4d). Notably, CM from *ATF2*-KD hUC-MSCs was less able to repress the secretion of TNF-α and IL-6 by LPS-stimulated MH-S cells than CM from control hUC-MSCs (Fig. 4d), indicating impairment of the anti-inflammatory potency of *ATF2*-KD cells. These results were validated further by examining the repressive effects of CM from the control and *ATF2*-KD cells on the expression levels of various proinflammatory genes in the LPS-treated MH-S cells (Fig. 4e and Supplementary Fig. 6).

Importantly, the ectopic expression of *ATF2* enhanced the PDGF-responsive chemotaxis activities in both hES- and hUC-MSCs (Supplementary Fig. 7a–e). In addition, the proangiogenic or anti-inflammatory capacities were increased by the overexpression of *ATF2* in hES- or hUC-MSCs, respectively (Supplementary Fig. 7c, f, and g). Collectively, the results of these in vitro functional assays indicate that ATF2 plays a critical role in preserving the primitive state of MSCs, as evidenced by their improved self-renewal, migratory, proangiogenic, anti-inflammatory, and immunomodulatory activities, all of which are related to their therapeutic potency.

### The role of GSH dynamics in the ATF2-dependent functionality of MSCs

**Fig. 5:**
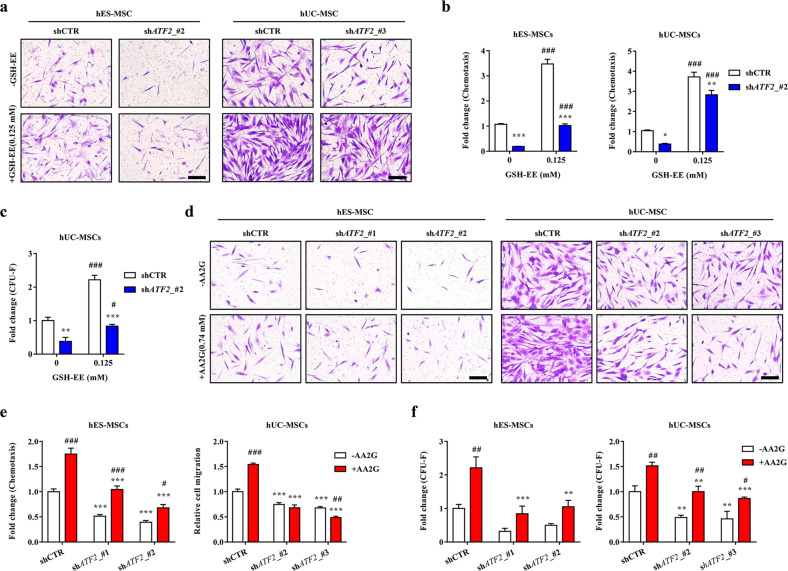
The role of GSH dynamics in the ATF2-dependent functionality of MSCs. Chemotaxis (**a** and **b**) in response to treatment with 10 ng/mL PDGF-AA (*n* = 7; **b**) and CFU-F potency (*n* = 3; **c**) in the control (shCTR) and *ATF2*-silenced (sh*ATF2*) MSCs with 0.125 mM GSH-EE. Chemotaxis (**d** and **e**) in response to treatment with 10 ng/mL PDGF-AA (*n* = 7; **e**) and CFU-F potency (*n* = 3; **f**) in the control (shCTR) and *ATF2*-silenced (sh*ATF2*) MSCs treated with 0.74 mM AA2G for 72 h. Representative images of the chemotaxis assays are presented on the left (magnification, ×200; scale bar, 100 µm). Quantitative data are represented as fold changes relative to the shCTR group and are displayed as the mean ± SEM. Statistical analyses were performed via two-way ANOVA (*p < 0.05, **p < 0.01, ***p < 0.001 compared with shCTR cells; #*p* < 0.05, ##*p* < 0.01, ###*p* < 0.001 compared with nontreated cells).

Next, we investigated whether the ATF2-mediated functionality of MSCs is dependent on the intracellular GSH level. To this end, *ATF2*-KD MSCs, which showed a reduced basal level of GSH and GRC (Fig. 2f), as well as impaired clonogenic and migratory capacities (Figs. 3 and 4), were treated with GSH-EE, a cell-permeable form of GSH. Notably, the defects in the potency of CFU-F (self-renewal) and PDGF-responsive chemotaxis capacity observed in *ATF2*-KD MSCs were rescued by treatment with GSH-EE (Fig. 5a−c).

This result led us to explore whether ATF2 is involved in the beneficial effects of GSH-enhancing priming by supplementation with AA2G. Consistent with previous reports^10,23^, AA2G priming increased PDGF-responsive migration and self-renewal (CFU-F potency) in both hES-MSCs and hUC-MSCs (Fig. 5d−f and Supplementary Fig. 8). These beneficial effects of AA2G were impaired significantly by silencing *ATF2*, indicating the critical role of ATF2 in the mode of action of AA2G priming of MSCs. As previously reported^23^, the PFO procedure based on AA2G supplementation increased the core functions of MSCs, including self-renewal (CFU-F), PDGF-responsive cell migration, and the proangiogenic, anti-inflammatory, and immunomodulatory capacities of MSCs. Importantly, the silencing of *ATF2* significantly impaired these beneficial effects of the PFO procedure in both hES-MSCs and hUC-MSCs (Supplementary Fig. 9), further demonstrating the importance of ATF2 in AA2G-based GSH-enhancing priming conditions. Overall, these results demonstrate the importance of ATF2 as a novel mediator of GSH dynamics and related primitiveness during ex vivo expansion and priming of MSCs.

### The importance of ATF2 for the use of MSCs in asthma therapy in vivo

**Fig. 6:**
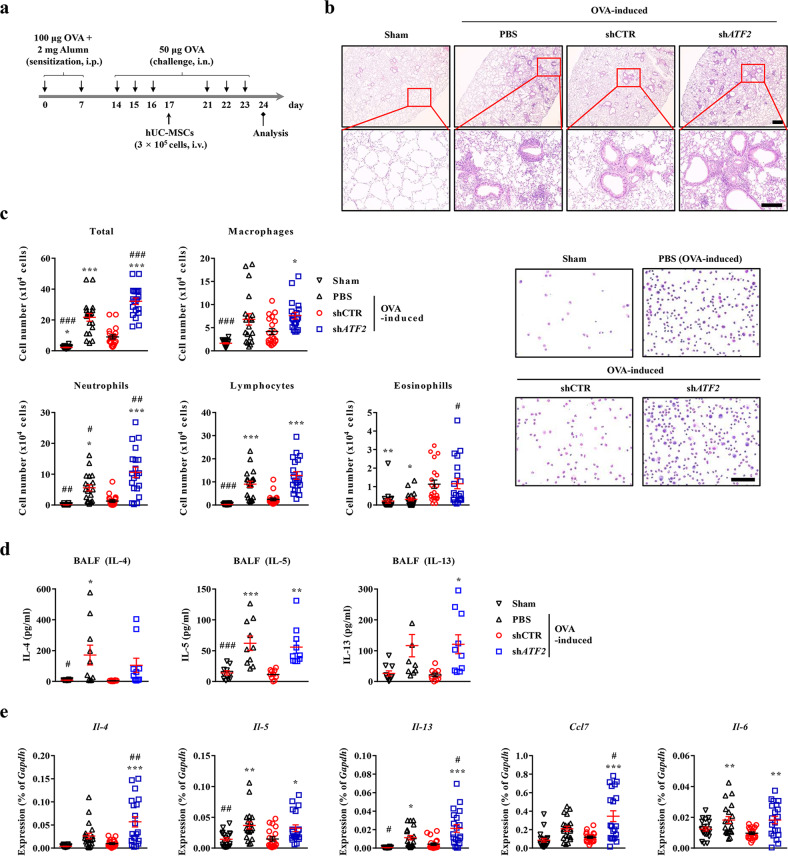
Silencing of *ATF2* impairs the therapeutic effects of MSCs in a murine model of allergic asthma. **a** Schematic overview of the experimental protocols for the induction of asthma and the intravenous (i.v.) injection of 3 × 10^5^ hUC-MSCs harboring a control (shCTR) or *ATF2*-specific shRNA (sh*ATF2*). OVA ovalbumin, alumn aluminum hydroxide, i.p. intraperitoneal injection, i.n. intranasal injection. **b** Hematoxylin and eosin staining of lung tissues (magnification, ×40, scale bar, 250 µm) from the sham mice and the OVA-induced mice injected with vehicle control (PBS) or hUC-MSCs expressing shCTR or sh*ATF2*. Higher magnification images (×200) are shown in the lower panels. Scale bar, 100 µm. **c** The numbers of total cells, macrophages, neutrophils, lymphocytes, and eosinophils identified via cytospin staining of BALF from mice (*n* = 20) in the indicated groups. Representative images of cytospin staining (magnification, ×400) are also shown. Scale bar, 50 µm. **d** Quantification of IL-4, IL-5, and IL-13 proteins in BALF from mice (*n* = 10) in the indicated groups. **e** RQ-PCR analyses of the indicated cytokines in lung tissues from mice (*n* = 20) in the indicated groups. Quantitative data are represented as the mean ± SEM. Statistical significance was examined via one-way ANOVA with Bonferroni *post hoc* tests (*p < 0.05, **p < 0.01, ***p < 0.001 compared with the shCTR group; #*p* < 0.05, ##*p* < 0.01, ###*p* < 0.001 compared with the PBS group). The exact *p* values and numbers of replicates are available in Supplementary Dataset 2.

To confirm the findings described above in vivo, we employed an OVA mouse model of allergenic asthma, which represents a Th2 immune cell-driven inflammatory airway allergic response^27^, and compared the therapeutic potencies of a single intravenous injection of 3 × 10^5^ hUC-MSCs expressing a control (shCTR) or *ATF2*-specific (sh*ATF2*) shRNA (Fig. 6a). As reported previously^33^, severe inflammation in the bronchial and vascular areas of the lung tissues was observed in the OVA-sensitized asthmatic mice administered PBS vehicle (Fig. 6b). Examination of the BALF revealed that these OVA-induced asthmatic mice displayed a significant increase in the overall cellularity and abundance of inflammatory cells (Fig. 6c). Single administration of hUC-MSCs expressing shCTR attenuated the inflammation of the lung tissue and infiltration of inflammatory cells, including macrophages, neutrophils, and lymphocytes, in the BALF (Fig. 6b, c). However, these beneficial effects were significantly defective in asthmatic mice injected with hUC-MSCs expressing sh*ATF2*.

Consistent with these findings, the levels of the IL-4, IL-5, and IL-13 proteins, all of which are Th2 immune response mediators, were lower in the BALF of asthmatic mice administered hUC-MSCs expressing shCTR than in that of asthmatic mice administered the vehicle control; however, this was not the case for mice administered hUC-MSCs expressing sh*ATF2* (Fig. 6d). The asthmatic mice were characterized by upregulation of genes related to the Th2-mediated immune response (e.g., *Il-4*, *Il-5*, and *Il-13*) and proinflammatory cytokines (e.g., *Ccl7*, *Il6*, *Tnf-α*, and *Inf-γ*); these increases were attenuated by single administration of hUC-MSCs expressing shCTR but not by administration of those expressing sh*ATF2* (Fig. 6e, Supplementary Fig. 10a, b). Overall, this preclinical study demonstrates that ATF2 plays a crucial role in MSCs to alleviate airway inflammatory responses.

**Fig. 7:**
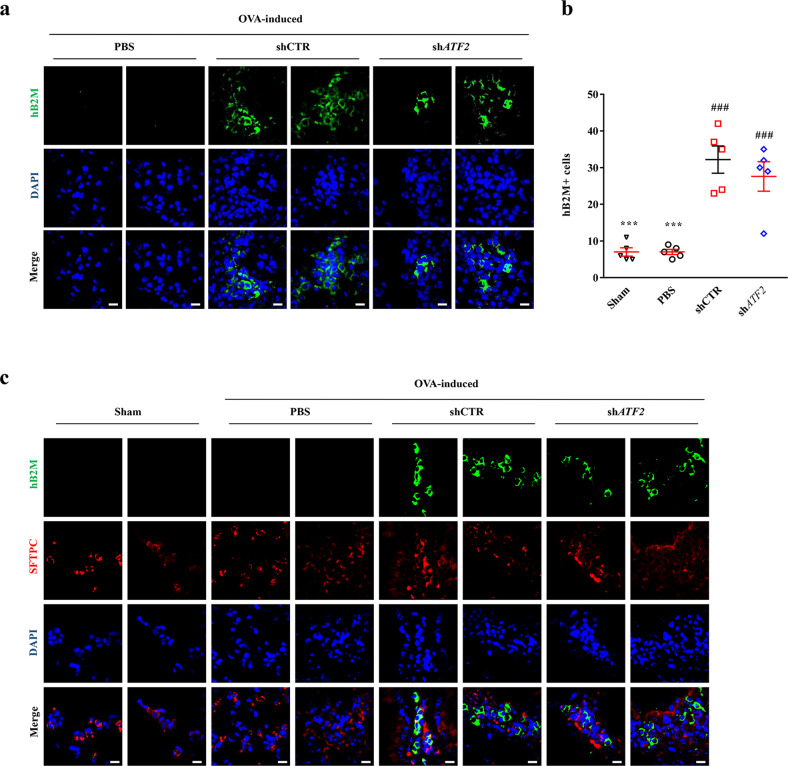
Immunostaining analysis of the engraftment and cellular properties of transplanted hUC-MSCs. **a** Immunostaining to detect cells expressing hB2M (green) in the lung tissues of OVA-stimulated asthmatic mice 1 week after injection of PBS vehicle or hUC-MSCs harboring a control (shCTR) or *ATF2*-specific (sh*ATF2*) shRNA (magnification, ×1000; scale bar, 200 µm). Lower magnification images (×200) are shown in Supplementary Fig. 10c. **b** Quantification of the engrafted hB2M^+^ cells in lung tissues from mice (*n* = 5) in the indicated groups. Quantitative data are shown as the mean ± SEM. Statistical significance was examined via one-way ANOVA with Bonferroni *post hoc* tests (***p < 0.001 compared with the shCTR group; ###*p* < 0.001 compared with the PBS group). **c** Representative confocal micrographs showing the immunohistochemical detection of hB2M (green) and the alveolar epithelial cell marker SFTPC (red) in lung tissues from mice in the indicated groups. Nuclei were stained with DAPI (blue). Two independent images are shown (magnification, ×1000; scale bar, 200 µm). Negative control experiments using mouse and rabbit IgG control antibodies are shown in Supplementary Fig. 10d.

Next, we examined whether ATF2 could modulate the in vivo engraftment of hUC-MSCs in the lung tissues of OVA-induced asthmatic mice. Staining of the lung tissues with an hB2M-specific antibody revealed that the frequencies of hB2M^+^ engrafted cells were comparable in the mice administered hUC-MSCs expressing shCTR and those administered hUC-MSCs expressing sh*ATF2* (Fig. 7a, b, and Supplementary Fig. 10c), indicating that ATF2 had a minimal effect on in vivo engraftment of the MSCs. Confocal microscopy analyses of the lung tissues revealed that the hB2M^+^ engrafted cells were not stained with an antibody targeting SFTPC, a type 2 alveolar epithelial cell marker. Instead, the majority of hB2M^+^ cells were located in proximity to SFTPC^+^ cells in both the shCTR and sh*ATF2* groups (Fig. 7c and Supplementary Fig. 10d). Therefore, these results indicate that the engrafted cells protected against the airway inflammation response via a paracrine effect rather than by directly contributing to tissue-resident cells.

We next investigated whether MSC priming with ATF2 activation could be beneficial for treating allergic asthma. To address this issue, we treated OVA-sensitized asthmatic mice with hES- or hUC-MSCs, which were expanded ex vivo by normal culture (naïve) or the PFO procedure. Compared with naïve MSCs, both MSCs by the PFO procedure showed superior therapeutic efficacy, based on the findings of the better attenuation of lung inflammation and the infiltration of inflammatory cells, particularly macrophages and neutrophils (Supplementary Figs. 11a–c and 12a–c). The frequencies of hB2M^+^ engrafted cells were increased in the lungs of mice administered PFO-primed MSCs (Supplementary Figs. 11d and 12d), indicating that the superior in vivo engraftment of PFO-MSCs could be responsible for their improved therapeutic potency compared with that of naïve MSCs.

## Discussion

GSH dynamics are important for preserving both the primitive state of MSCs and their therapeutic efficacy toward several intractable disorders^9,21,23,34^. The results presented here demonstrate that ATF2 is a novel mediator of GSH dynamics in MSCs that acts via interplay with the NRF2 signaling cascade, thereby affecting the functionality and therapeutic potency of MSCs toward allergic asthma.

Several reports have described protocols for the stable preservation and ex vivo expansion of primitive MSCs with high therapeutic potency, for example, by enriching small-sized cells or enhancing the antioxidant capacity^9,10,20,21,23^. In these previous reports, transcriptome analyses revealed that MSCs with high levels of GSH displayed common molecular features, including activation of the CREB1-NRF2 pathway, resulting in the upregulation of genes related to GSH synthesis and redox cycling, as well as high expression levels of AP1 family transcription factors such as ATF2, JUN, JUNB, and FRA1. In our recent study, we found that the FOS proto-oncogene AP1 protein played a central role in maintaining both the core functions of hES-MSCs in vitro and the in vivo engraftment of transplanted hESC-MSCs, thus affecting their therapeutic potency in a preclinical study of interstitial cystitis/bladder pain syndrome^16^. AP1 activity is responsive to extracellular signals^29^, and the functions of AP1 complexes are diverse due to their ability to form distinct heterodimers. For example, ATF2 reportedly forms eight different complexes with other members of the ATF, JUN, and FOS families, whereas JUN can form 15 different dimeric complexes. Therefore, investigating the role of the interplay between these AP proteins in controlling GSH dynamics could not only advance our understanding of the molecular signature of the primitiveness of MSCs but also overcome the technical limitations of current MSC therapies.

Here, we found that ATF2 is a crucial mechanomediator of redox homeostasis in hES-MSCs and hUC-MSCs that acts by collaborating with the CREB1-NRF2 pathway. This finding is consistent with a previous report demonstrating that the ATF2 protein forms complexes with NRF2 and other multiple basic-leucine zipper proteins and is recruited to promote the heme oxygenase-1 gene following arsenite treatment^35^. In addition, another study found that ectopic expression of *ATF2* inhibited ferroptosis induced by a bromodomain and extraterminal domain protein inhibitor in human breast cancer cells by upregulating NRF2. The clinical relevance of the positive correlation between ATF2 and NRF2 was demonstrated via The Cancer Genome Atlas Program (TCGA) dataset analysis of breast, lung, and cervical tissues^36^. ATF2 is a stress-response protein that is upregulated by oxidative, inflammatory, and DNA damage stresses^29^. In esophageal squamous epithelial cancer cells, activation of ATF2 via phosphorylation of threonine residues 69 and 71 reduces oxidative stress-induced apoptosis and consequently reinforces cell cycle arrest by upregulating p21^WAF1^ and JUN^37^. In addition, resveratrol (3,5,4’-trihydroxystilbene), a naturally occurring polyphenol with antioxidant activity, reportedly increases the transcriptional activation potentials of CREB and ATF2 to mediate cytoprotective and tumor suppressive outcomes^38^. Therefore, the activation of ATF2 by diverse extracellular stimuli could affect the intracellular level and dynamics of GSH to modulate various biological processes, including inflammation, aging, tumorigenesis, and the primitiveness of stem cells.

In our current study, the expression level and activity of ATF2 were stimulated by priming human MSCs with AA2G, a VitC derivative, to enhance GSH dynamics. In our previous study^10^, we found that AA2B stably promoted the primitive state of MSCs and the naïve pluripotency of murine ESCs and overcame the critical drawbacks of VitC, which is extremely unstable in aqueous solution because it readily oxidizes to dehydroascorbate, leading to cellular toxicity. We also found that AA2G reproduced the known biological effects of VitC, including TET-dependent DNA demethylation in murine ESCs and suppression of p53 during the generation of murine iPSCs, and that activation of the CREB1 pathway accounted for the beneficial effects of AA2G in ESCs and MSCs^10^. Furthermore, priming with AA2G promoted the core functions of MSCs, including self-renewal (based on CFU-F activity), PDGF-responsive cell migration, and anti-inflammatory potency. The in vivo importance of these findings was demonstrated by using a polyinosinic:polycytidylic acid (poly-I:C)-induced murine asthma model representing viral infection pathogenesis^10^. In this study, ATF2 played a crucial role in the beneficial effects of the PFO procedure based on the combination of three small molecules, AA2G, S1P, and VPA, which significantly improved the therapeutic potency of MSCs from different sources for treating allergic asthma. In this regard, the present study elucidates a novel mode of action of various AA2G-based priming procedures, namely, the role of ATF2 in preserving GSH dynamics and the related primitiveness of MSCs. Therefore, we postulate that ATF2 might be responsible for the effects of AA2G priming in other populations of stem cells, such as human PSCs, neural stem cells, and hematopoietic stem cells. Further studies are required to verify this hypothesis. In addition, the direct target(s) or critical mediator(s) of ATF2 in controlling GSH dynamics should be identified in different cellular or microenvironmental contexts.

GSH-enhancing priming conditions, such as AA2G priming and the PFO procedure, could connect cellular redox signaling via numerous common pathways, such as the receptor tyrosine kinase and G protein-coupled receptor (GPCR) pathways^39^. GPCRs activate heterotrimeric G proteins in the plasma membrane; unlike the Gi (Gαi/o) subunit, the Gs alpha subunit protein (Gαs) is responsible for stimulating cAMP- and PKA-dependent pathways by activating adenylyl cyclase^39^. Notably, we previously found that the beneficial effects of AA2G priming in murine ESCs and human MSCs were prevented by treatment with melittin, which inhibits Gαs and stimulates Gαi/o, underlining the critical role of Gαs in AA2G priming-mediated effects. Furthermore, the GPCR-related genes *GNAI1* and *HTR2B* were identified as components of the CREB1-associated gene networks generated to characterize the transcriptome of AA2G-treated murine ESCs and human MSCs^10^. Therefore, GPCR signaling could play a crucial upstream role in modulating ATF2-mediated GSH dynamics and the related functionality of MSCs following AA2G priming. Additional studies are required to investigate the importance of GPCRs in the ATF2-NRF2 pathway, focusing on the specific roles of Gα subunit proteins in MSCs under different ex vivo expansion conditions.

Since the burden of uncontrolled asthma is substantial and growing continuously^40^, the identification of pathway-specific approaches for the prevention and treatment of this disease is required to reduce costs and improve the quality of life of patients. Due to their strong anti-inflammatory and immunomodulatory effects on innate and adaptive immune cells, MSCs have been used to treat intractable asthma, which is a major cause of morbidity and mortality worldwide^1,5^. In preclinical studies using animal models representing different pathogeneses, including asthma caused by house dust mites, poly-I:C, or OVA stimulation, MSC therapy was effective in alleviating airway inflammatory responses, hyperresponsiveness, and remodeling^7^. In our current study, we used an OVA-stimulated murine asthma model to demonstrate the in vivo importance of ATF2 in the therapeutic potency of MSCs, particularly toward airway inflammation, which is an important pathophysiological feature of asthma. The results of this preclinical study are consistent with our previous studies showing that AA2G-primed MSCs or those with high levels of GSH exhibit enhanced therapeutic potency in a mouse model of virus-associated asthma^9,10^.

In these previous preclinical studies, the in vivo engraftment capacity of MSCs with high GSH dynamics was superior to that of control MSCs. In contrast, *ATF2*-silenced MSCs were engrafted into the lung at considerably higher levels than control MSCs. To further examine this unexpected finding, we investigated the properties and locations of the engrafted cells by costaining the hB2M and SFTPC proteins. When hES-MSCs were sorted based on the intracellular level of GSH, the MSCs that survived in the lungs expressed the SFTPC protein, indicating their direct contribution to the alveolar epithelium^9^. Although MSCs reportedly take on the gene expression profile of lung epithelial cells both in vitro and in vivo^41–47^ and are stimulated by tissue injury^42^, the transdifferentiation of mesodermal MSCs into surfactant protein-producing cells is rare in a normal physiological environment. In this regard, our previous study showed that AA2G-primed hES-MSCs engrafted into mouse lungs showed little expression of SFTPC^10^. Similarly, in our current study, we found that the hB2M^+^ engrafted cells were negative for SFTPC expression but were located in the proximity of SFTPC^+^ type 2 alveolar epithelial cells. Importantly, the anti-inflammatory capacity of hUC-MSCs was severely impaired by *ATF2* silencing but enhanced by *ATF2* overexpression. Taken together, these findings indicate that the MSCs engrafted into mouse lungs induced an anti-inflammatory response via a paracrine effect rather than by directly transdifferentiating into tissue-resident cells. The anti-inflammatory and immunosuppressive activities of MSCs are mediated by cell contact-dependent mechanisms involving B7-H1^48^ and by the secretion of soluble factors such as IL-10, transforming growth factor-β, nitric oxide, prostaglandin E2, and indoleamine 2,3-dioxygenase^49,50^. Therefore, for determination of the mode of action of MSC therapies in asthma, further studies are required to investigate which mediators could be affected by ATF2.

In summary, this study demonstrates that ATF2 mediates GSH dynamics and the related functional and therapeutic ability of MSCs to alleviate inflammatory responses in an experimental asthma model. Moreover, this study provides an in vivo proof of concept that the expression or activity of ATF2 can be used as a biomarker for predicting and evaluating the functions of MSCs for their ex vivo expansion and therapeutic applications.

## Supplementary information

Supplementary Information

Source dataset
