# Supplementary material for: Activating transcription factor-2 supports the antioxidant capacity and ability of human mesenchymal stem cells to prevent asthmatic airway inflammation
Source: Exp Mol Med. 2023 Feb 10;55(2):413–25. doi: 10.1038/s12276-023-00943-z (PMC9981582; doi:10.1038/s12276-023-00943-z)
Supplement: Supplementary file 1 — Supplementary Information [file 12276_2023_943_MOESM1_ESM.pdf]

SUPPLEMENTARY INFORMATION

Activating transcription factor-2 supports the antioxidant capacity and ability of human mesenchymal stem cells to prevent asthmatic airway inflammation

Hyein Ju[1], HongDuck Yun[1], YongHwan Kim, Yun Ji Nam, Seungun Lee, Jinwon Lee, Seon Min Jeong, Jinbeom Heo, Hyungu Kwon, You Sook Cho, Gowun Jeong, Chae-Min Ryu[*], Dong-Myung Shin[*]

[1]These authors contributed equally to this work.

*Corresponding authors: d0shin03@amc.seoul.kr and chaemin0427@amc.seoul.kr

This PDF file includes:

Supplementary Fig. 1–12 and figure legends

Supplemenatry Tables 1–2

Supplementary references

Uncropped western blot results

Other supplementary information includes the following (separate file):

Supplementary Dataset 1. Values for the GSH dynamics index for each plot

Supplementary Dataset 2. Source data for quantification analyses

SUPPLEMENTARY FIGURE LEGENDS

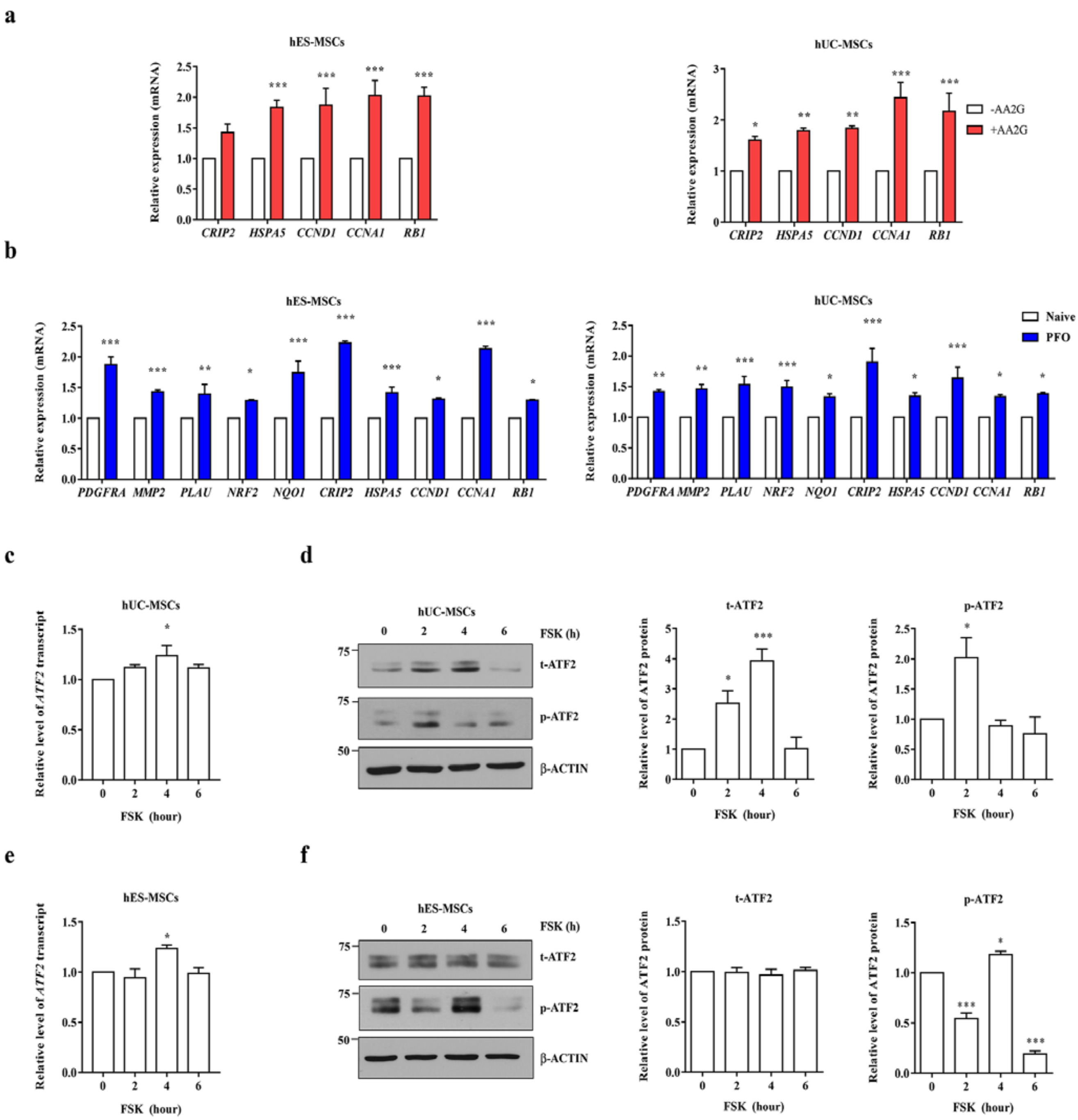

Supplementary Fig. 1 Activation of ATF2 by AA2G and forskolin priming in human MSCs

(a and b) RQ-PCR analyses (*n* = 4) of ATF2 target genes in hES-MSCs and hUC-MSCs

following treatment with 0.74 mM AA2G for 72 hours **(a)** or the PFO procedure **(b)** culture. The PFO procedure included the supplementation of 0.74 mM AA2G for two days, followed by further stimulation with 50 nM sphingosine 1-phosphate (S1P) and 0.5 mM valproic acid (VPA). **(c–f)** RQ-PCR analyses (n = 4) of the *ATF2* transcript **(c and e)** and western blot analyses (n = 3) of total (t-ATF2) and phosphorylated ATF2 (p-ATF2) proteins **(d and f)** in FSK-treated hUC-MSCs **(c and d)** and hES-MSCs **(e and f)**. The expression level of β-actin was used as a loading control for western blot analysis. Molecular weight marker sizes (kD) are shown on the left of the blots. All quantification results are shown as the mean ± SEM (*$p < 0.05$, **$p < 0.01$, ***$p < 0.001$ compared with nontreated cells, via one-way **(b–e)** or two-way **(a)** ANOVA with Bonferroni *post hoc* tests).

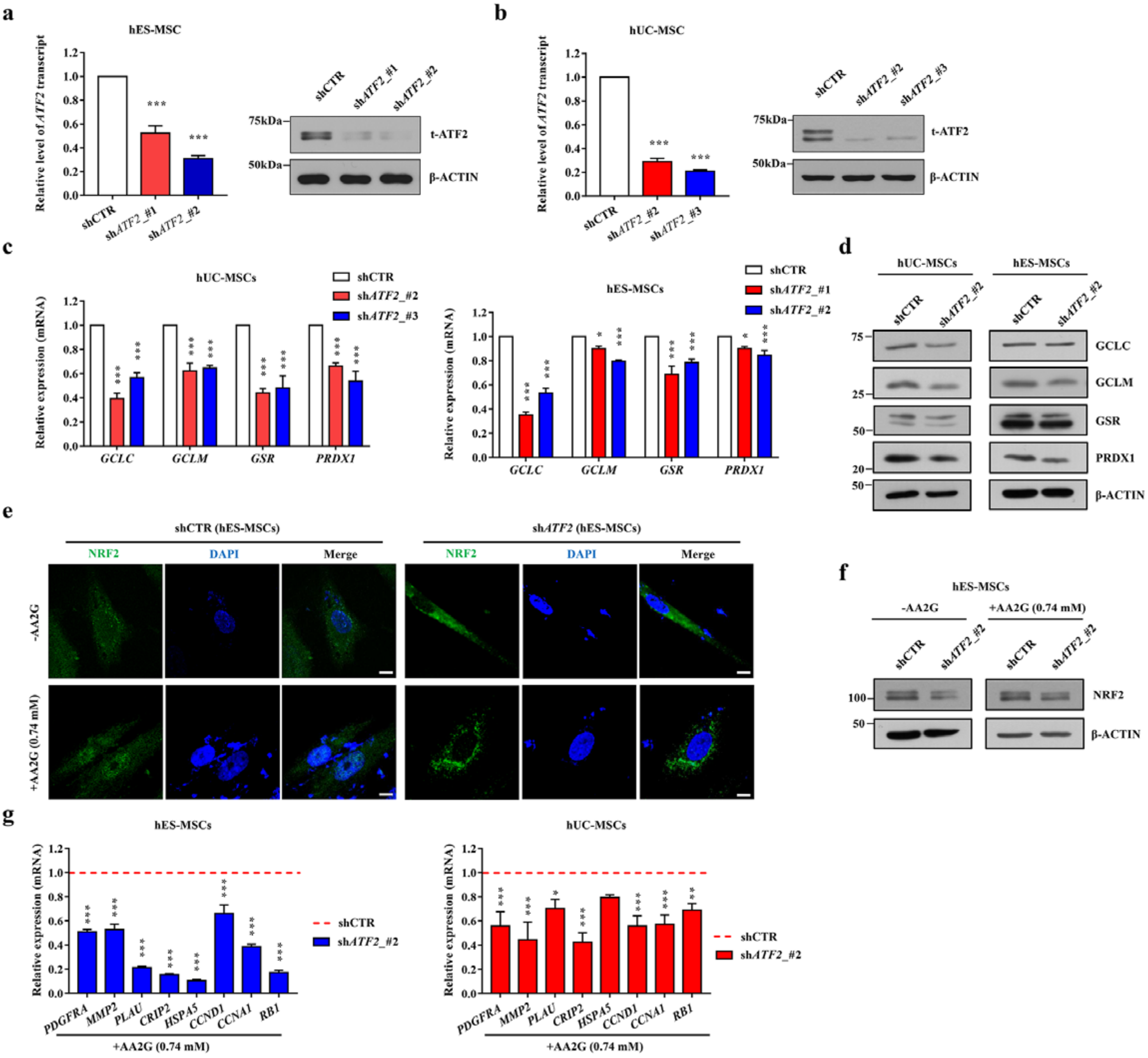

Supplementary Fig. 2 Repression of NRF2 target genes by silencing of *ATF2* in human MSCs

(a and b) RQ-PCR analyses of the *ATF2* transcript and western blot analyses of the ATF2 protein in hES-MSCs **(a)** and hUC-MSCs **(b)** harboring a scrambled (shCTR) or *ATF2*-specific (sh*ATF2*) shRNA. Two independent sh*ATF2* constructs were used. The expression level of β-

actin was used as a loading control for western blot analysis. Molecular weight marker sizes (kD) are shown on the left of the blots. **(c and d)** RQ-PCR **(c)** and western blot **(d)** analyses of NRF2 target genes in control and *ATF2*-silenced MSCs. **(e)** Representative confocal microscopy images of the NRF2 protein (green) in hES-MSCs treated with or without AA2G and expressing shCTR or sh*ATF2*. Nuclei were stained with DAPI (blue). Magnification, ×1,000. Scale bar, 10 µm. **(f)** Western blot analyses of NRF2 protein in MSCs expressing a scrambled control shRNA (shCTR) or an *ATF2*-specific shRNA (sh*ATF2*) with or without AA2G treatment. **(g)** RQ-PCR analyses of ATF2 target genes in control and *ATF2*-silenced MSCs. Quantitative data are represented as fold changes relative to the shCTR group (set to 1; see the red dotted line in **g**), and are displayed as the mean ± SEM (n = 4; *p < 0.05, **p <0.01, ***p < 0.001 compared with the shCTR group, via one-way **(a and b)** or two-way **(c and g)** ANOVA with Bonferroni *post hoc* tests).

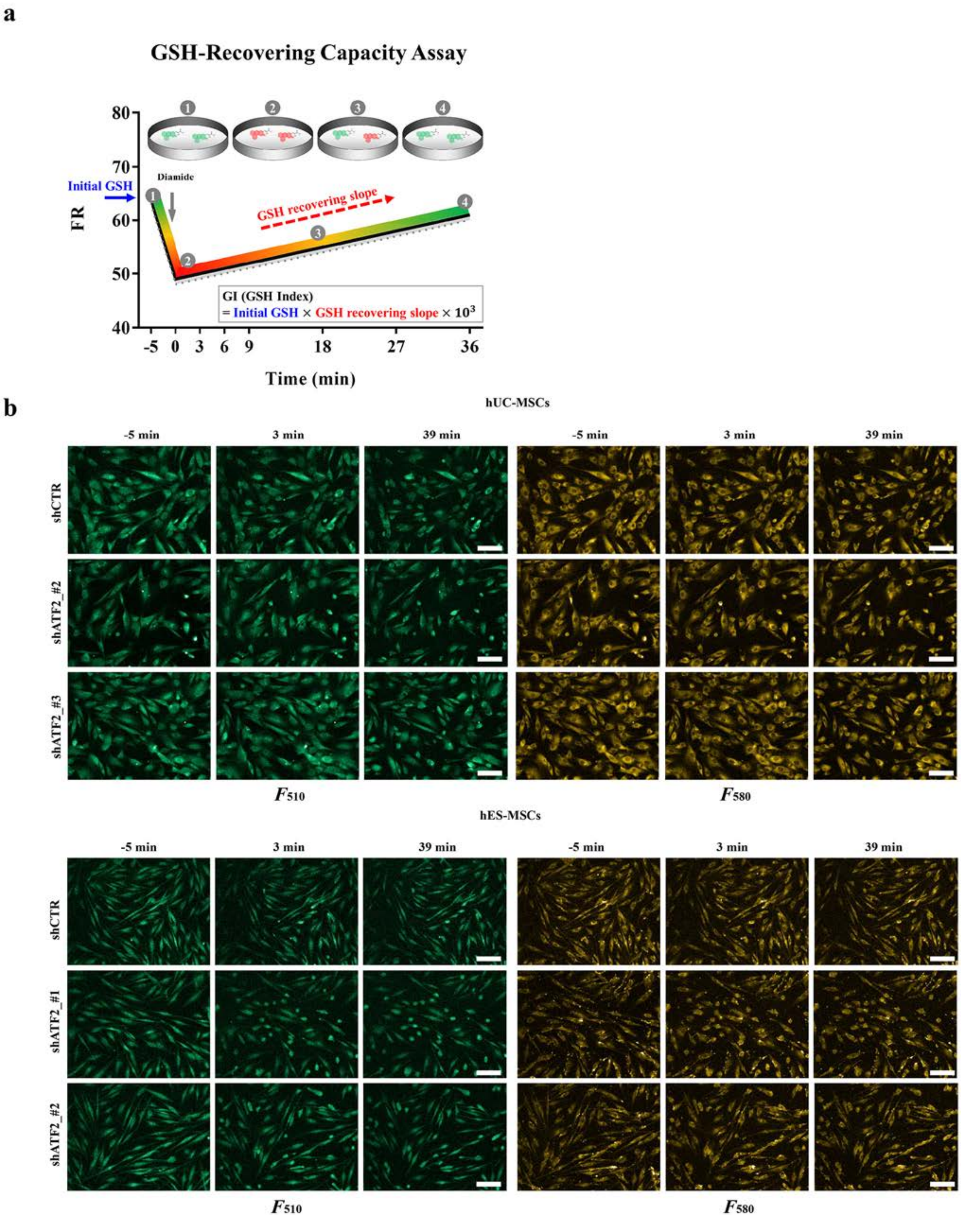

Supplementary Fig. 3 Real-time live-cell GSH recovery capacity assay

(a) A schematic overview for evaluating the GSH recovery capacity (GRC), an indicator of cellular antioxidant capacity. Changes in GSH dynamics in every living single cell were monitored in real-time using an Operetta High-Content Imaging System and FreSH tracer (Fluorescent real-time thiol tracer), a reversible GSH fluorescent probe [1,2]. The GI of each sample following exposure to 0.1 or 0.2 mM diamide was quantified based on both the initial F_{510}/F_{580} fluorescence ratio (FR) (representing the baseline total GSH) and the slope after diamide treatment (representing the GRC), as described previously [2]. **(b)** Representative images of F_{510} (GSH bound) and F_{580} (GSH free) fluorescence in hES-MSCs and hUC-MSCs carrying the indicated shCTR or sh*ATF2* constructs. The related FR plots with quantification results are shown in **Fig. 2f**.

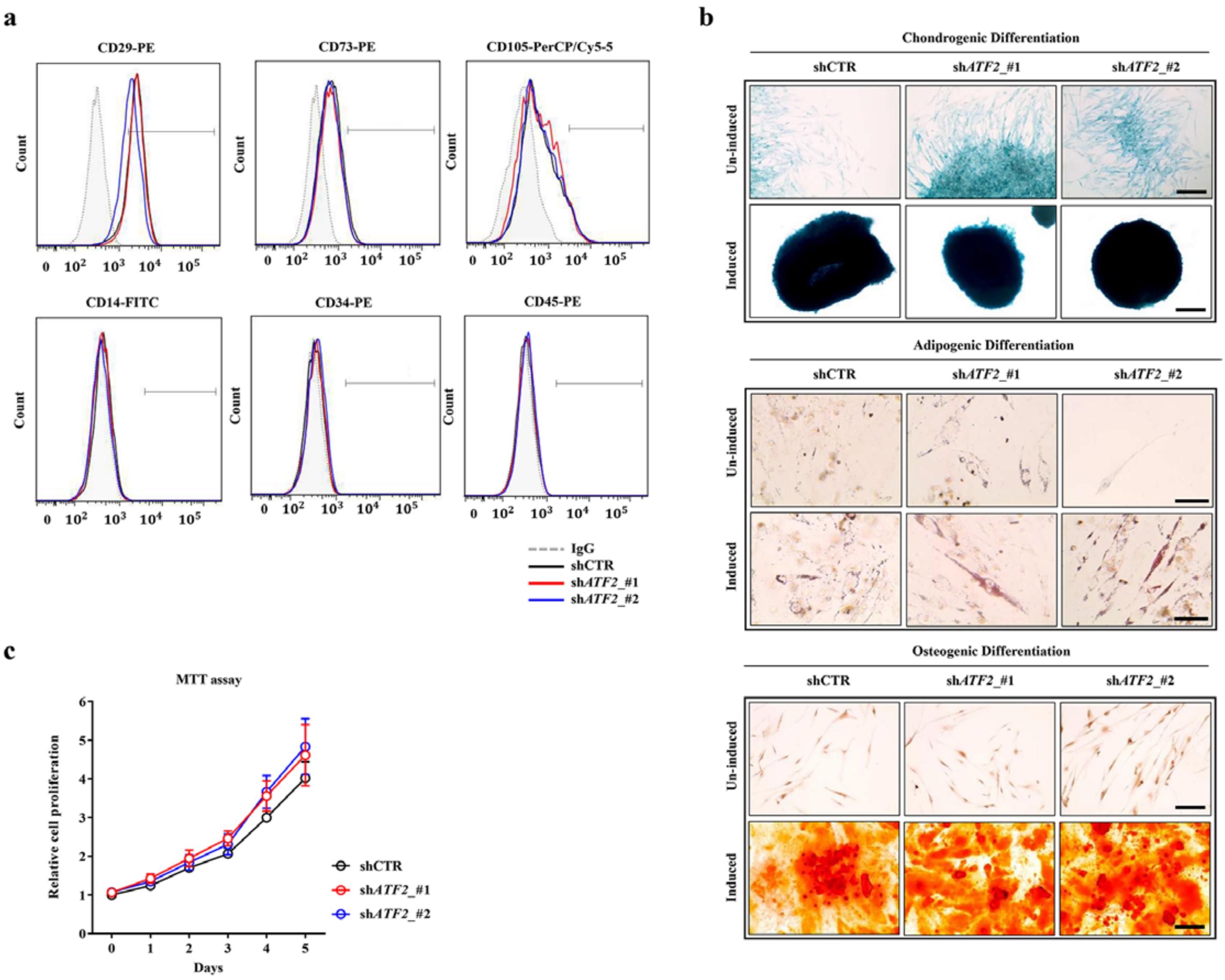

Supplementary Fig. 4 The effect of silencing *ATF2* on the basic functions of hES-MSCs

(a) Flow cytometry analyses of the expression of MSC surface proteins (CD29, CD73, and CD105) and hematopoietic lineage markers (CD14, CD34, and CD45) in hES-MSCs harboring scrambled control (shCTR) or *ATF2*-specific (sh*ATF2*) shRNA constructs. An IgG isotype antibody (dotted line) was used as a control. **(b)** Differentiation of control and *ATF2*-KD hES-MSCs into chondrogenic (top panel; magnification, ×100; scale bar, 200 μm), adipogenic (middle panel; magnification, ×400; scale bar, 50 μm), and osteogenic (bottom panel; magnification, ×200; scale bar, 200 μm) lineages, determined using Alcian Blue, Oil Red O,

and Alizarin Red S staining, respectively. **(c)** The proliferation activities of control and *ATF2*-KD hES-MSCs, as determined by an MTT assay on the indicated days. Data are shown as fold changes relative to the control group (shCTR) and are displayed as the mean ± SEM (n = 4).

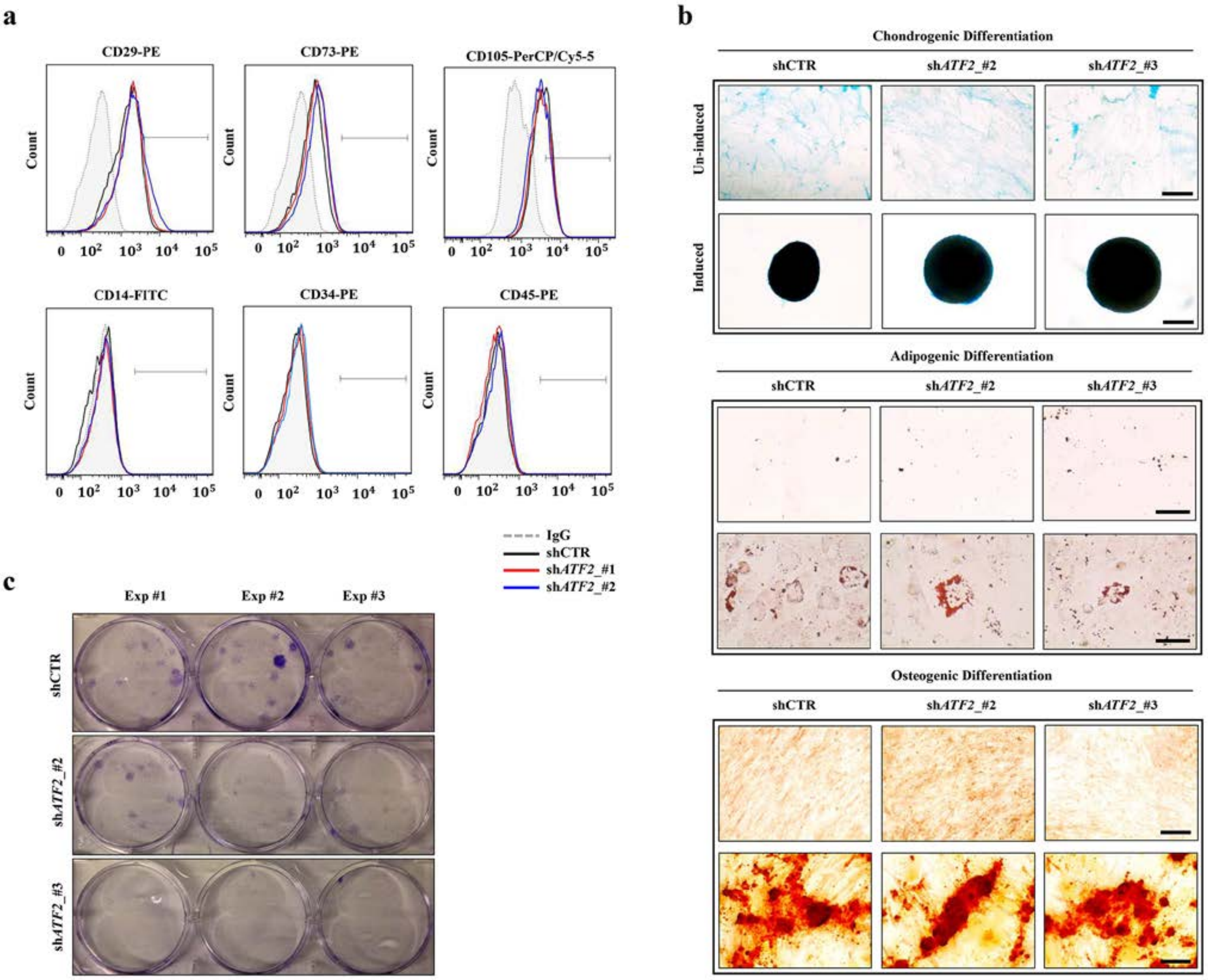

Supplementary Fig. 5 The effect of silencing *ATF2* in hUC-MSCs

(a) Flow cytometry analysis of the expression of MSC surface proteins (CD29, CD73, and CD105) and hematopoietic lineage markers (CD14, CD34, and CD45) in hUC-MSCs harboring scrambled control (shCTR) or *ATF2*-specific (sh*ATF2*) shRNA constructs. An IgG isotype antibody (dotted line) was used as a control. **(b)** Differentiation of control and *ATF2*-KD hUC-MSCs into chondrogenic (top panel; magnification, ×100; scale bar, 200 μm), adipogenic (middle panel; magnification, ×400; scale bar, 50 μm), and osteogenic (bottom panel; magnification, ×100; scale bar, 200 μm) lineages, determined using Alcian Blue, Oil

Red O, and Alizarin Red S staining, respectively. **(c)** Representative images of colony forming unit-fibroblast (CFU-F) assays in control and *ATF2*-KD hUC-MSCs.

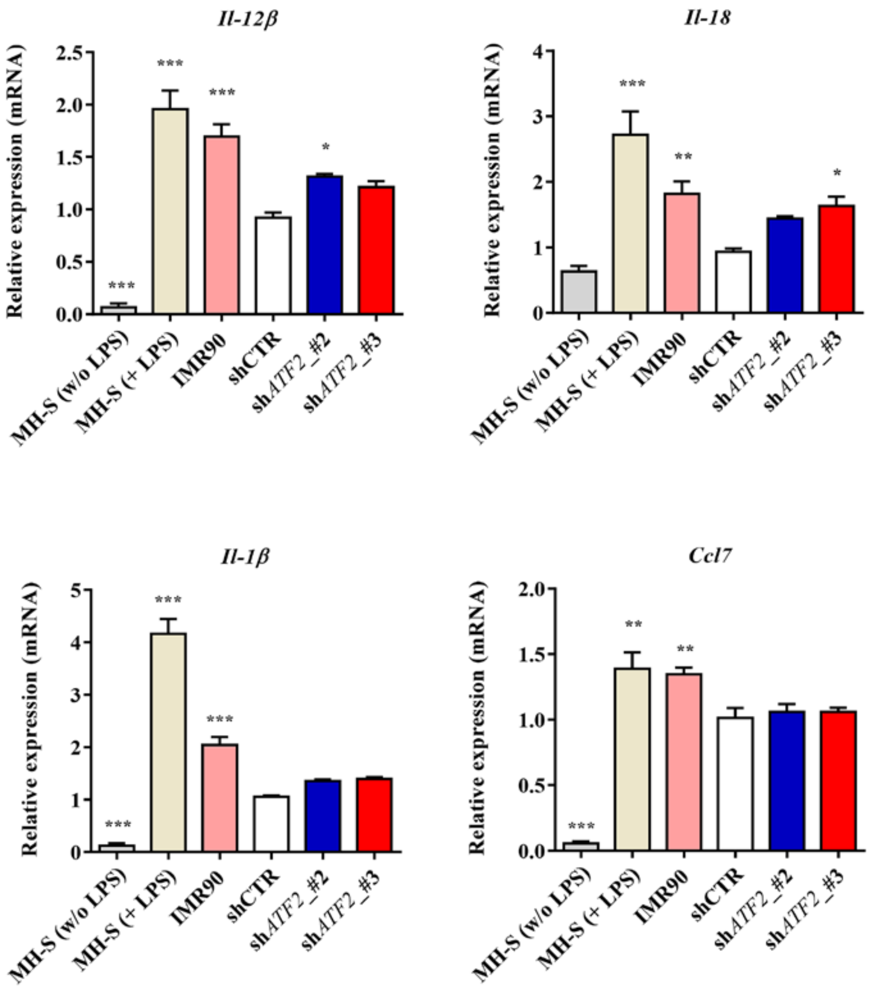

Supplementary Fig. 6 The anti-inflammatory activity of *ATF2*-silenced hUC-MSCs

RQ-PCR analyses of proinflammatory genes in MH-S cells that were pretreated with lipopolysaccharide (LPS) and then incubated with conditioned medium (CM) from the indicated cells. CM from IMR90 normal primary fibroblasts was used as a control. The expression levels of the indicated genes are represented as fold changes relative to the shCTR group and are shown as the mean ± SEM (n = 6; *p < 0.05; **p < 0.01; ***p < 0.001 compared with the shCTR group, via a one-way ANOVA with Bonferroni *post hoc* test).

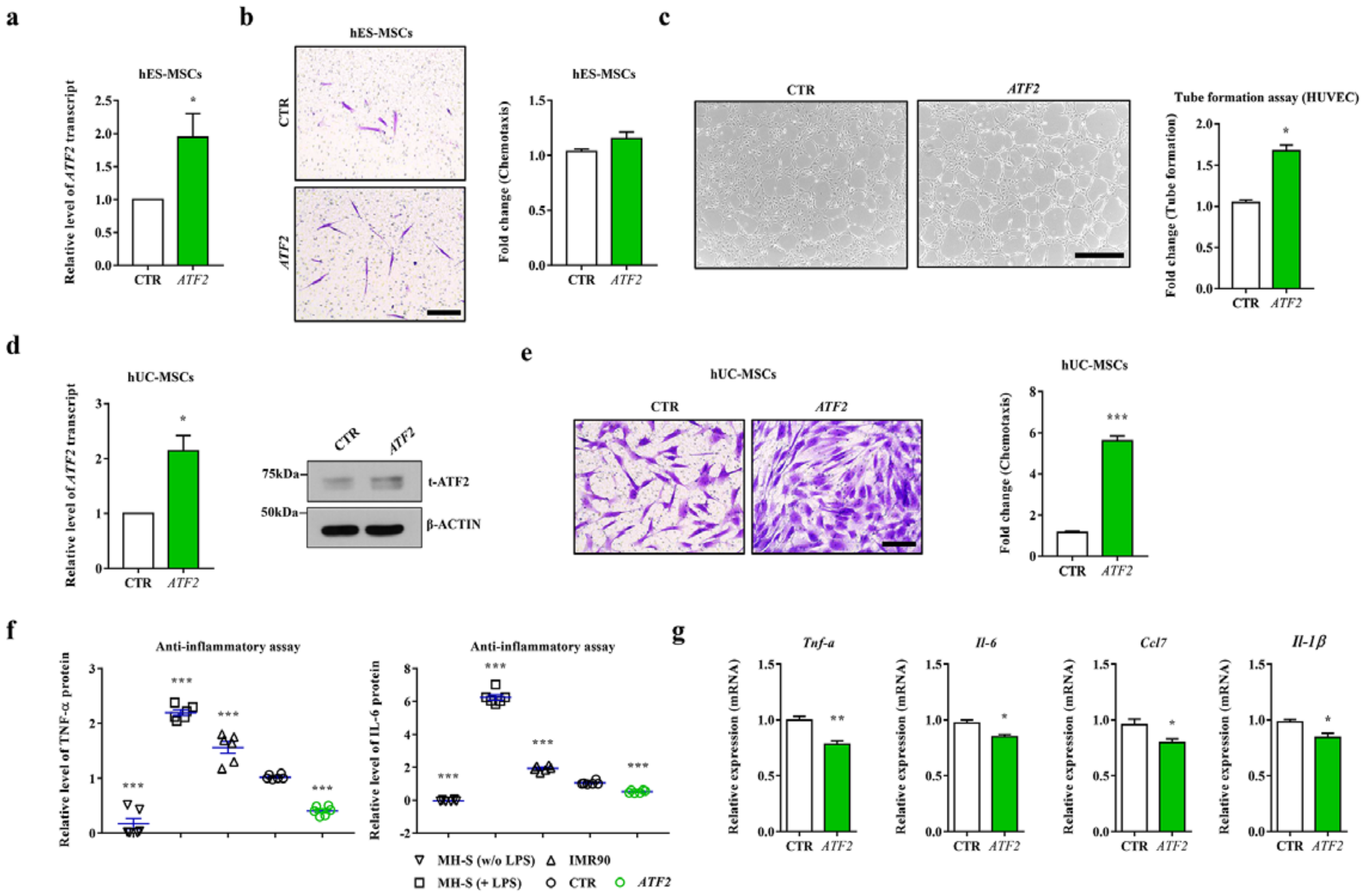

Supplementary Fig. 7 Ectopic expression of *ATF2* enforces the core functions of MSCs

(a) RQ-PCR (n = 4) analyses for the over-expression of *ATF2* in hES-MSCs. **(b and c)** The PDGF-responsive chemotaxis (n = 7; **b**) and pro-angiogenesis (n = 4; **c**) activities in hES-MSCs harboring an empty control (CTR) or human *ATF2* ORFs. **(d)** RQ-PCR (n = 4) and western blot analyses for the over-expression of *ATF2* in hUC-MSCs. **(e)** The PDGF-responsive chemotaxis (n = 7) activity in hUC-MSCs over-expressing *ATF2*. **(f and g)** Anti-inflammation assays via examining the inhibitory effects of the conditioned medium (CM) from hUC-MSCs after the stimulation with lipopolysaccharide (LPS) in MH-S cells. **(f)** Quantification of TNF-α and IL-6 proteins (n = 6) from the LPS-stimulated MH-S cells by an ELISA assay. **(g)** RQ-PCR analysis of the proinflammatory genes in MH-S cells that were pretreated with the CM

from the indicated cells (n = 6). The expression levels of the indicated genes are represented as fold changes relative to the CTR group and are shown as the mean ± *SEM*. *p < 0.05; **p < 0.01; ***p < 0.001, compared with the CTR group, via nonparametric Mann-Whitney *U* tests **(a–e and g)** or one-way ANOVA with Bonferroni *post hoc* test **(f)**.

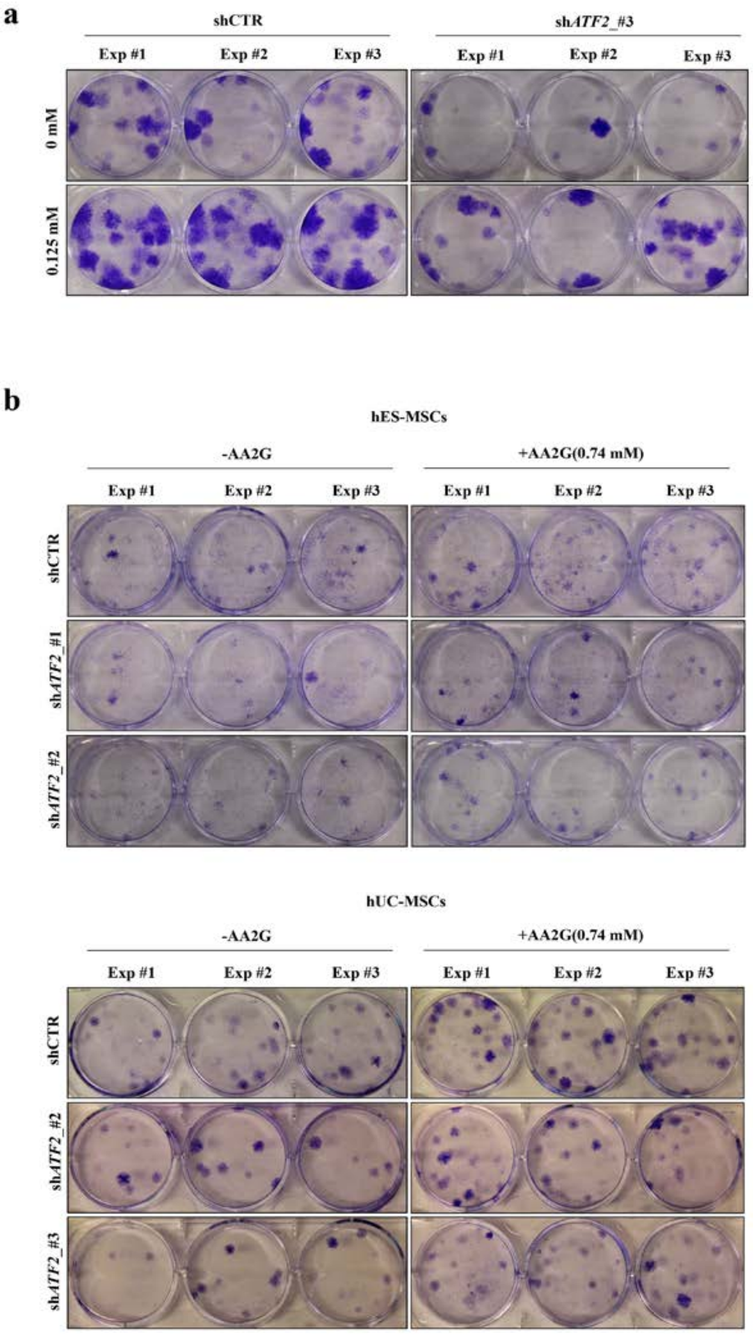

Supplementary Fig. 8 The self-renewal activity of *ATF2*-silenced MSCs supplemented with GSH

(a and b) Representative images of CFU-F assays of control and *ATF2*-silenced MSCs treated with 0.125 mM GSH-EE, a cell-permeable form of GSH, for 4 hours **(a)**, or with 0.74 mM AA2G for 72 hours **(b)**. The related quantification results are shown in **Fig. 5c and 5f**.

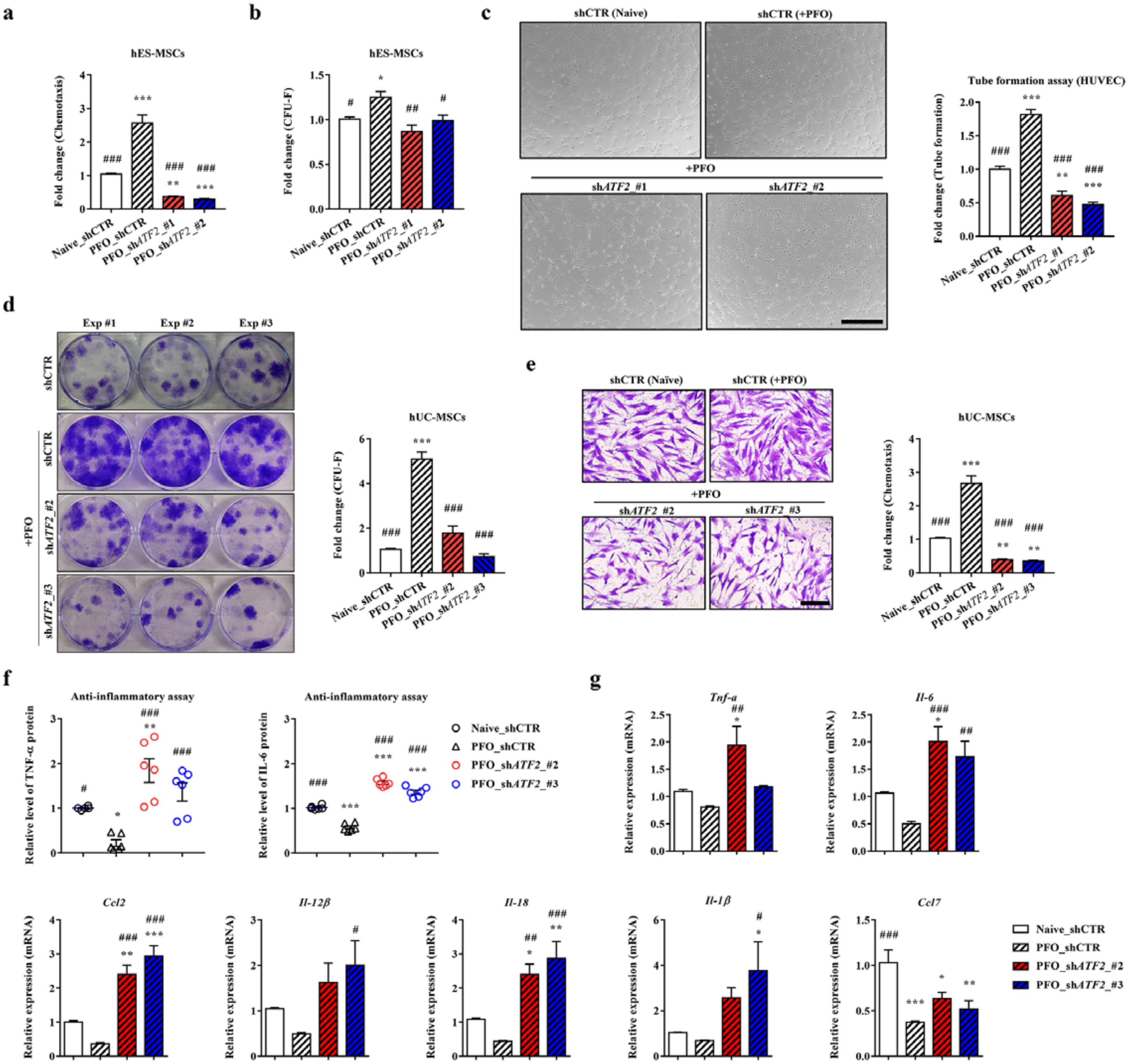

Supplementary Fig. 9 Role of ATF2 on the beneficial outcomes of the PFO procedure

(a–c) Analyses of CFU-F (n = 3; **a**), PDGF-responsive chemotaxis (n = 7; **b**), and pro-angiogenesis (n = 4; **c**) activities in hES-MSCs after naïve culture or subjection to the PFO procedure with the supplement of AA2G for two days. **(d–g)** Analyses of CFU-F (n = 3; **d**), PDGF-responsive chemotaxis (n = 7; **e**), quantification of TNF-α and IL-6 proteins as anti-

inflammation assays with lipopolysaccharide (LPS) and then incubated with conditioned medium (CM) (n = 6; **f**), and RQ-PCR analyses (n = 6; **g**) of proinflammatory genes in MH-S cells that were pretreated with the CM from the indicated cells. The expression levels of the indicated genes are represented as fold changes relative to the not treated (Naïve) shCTR group and are shown as the mean ± *SEM* (*p < 0.05; **p < 0.01; ***p < 0.001, compared with the naïve shCTR group; #p < 0.05; ##p < 0.01; ###p < 0.001, compared with the PFO shCTR group, via a one-way ANOVA with Bonferroni *post hoc* test)

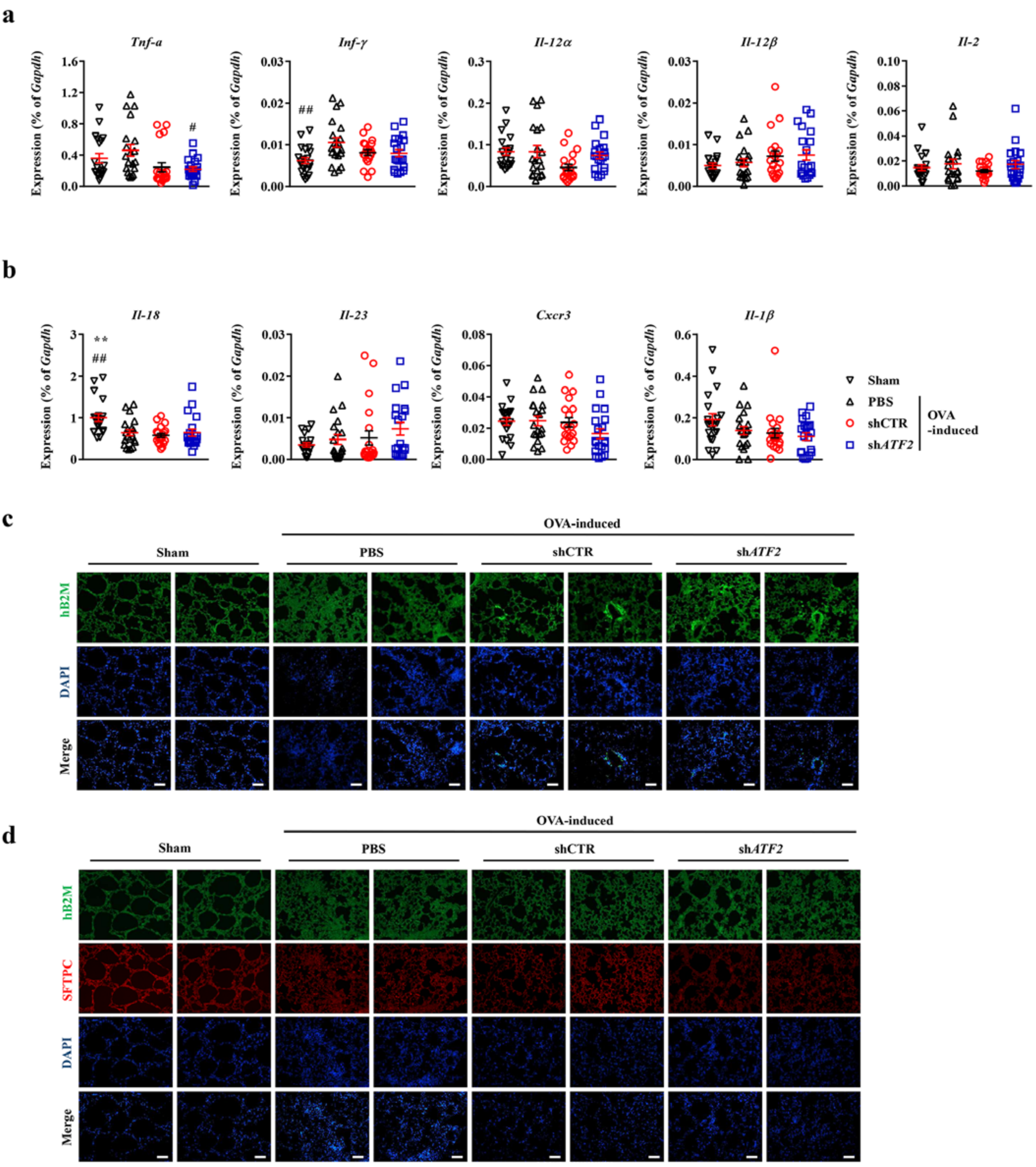

Supplementary Fig. 10 Cytokine profiles and the engraftment of MSCs in the lungs of asthmatic mice

(a and b) Expression of cytokine genes in the lungs of asthmatic mice. RQ-PCR analyses of

genes related to proinflammatory **(a)** and Th1 **(b)** immune responses in the lungs of asthmatic mice 1 week after injection of PBS vehicle or hUC-MSCs harboring a control (shCTR) or *ATF2*-specific (sh*ATF2*) shRNA (n = 20). **(c and d)** Immunostaining of transplanted hUC-MSCs. **(c)** Representative images of immunostaining (magnification, ×200) to detect hB2M (green) in the lung tissues of OVA-stimulated asthmatic mice administered PBS vehicle or hUC-MSCs harboring a control (shCTR) or *ATF2*-specific (sh*ATF2*) shRNA. Scale bar, 200 µm. **(d)** To rule out the possibility of nonspecific staining, lung tissues of the OVA-induced asthmatic mice were costained with mouse and rabbit IgG control antibodies for hB2M (green) and the alveolar epithelial cell marker SFTPC (red) as negative controls (magnification, ×200; scale bar, 200 µm. Nuclei were stained with DAPI (blue).

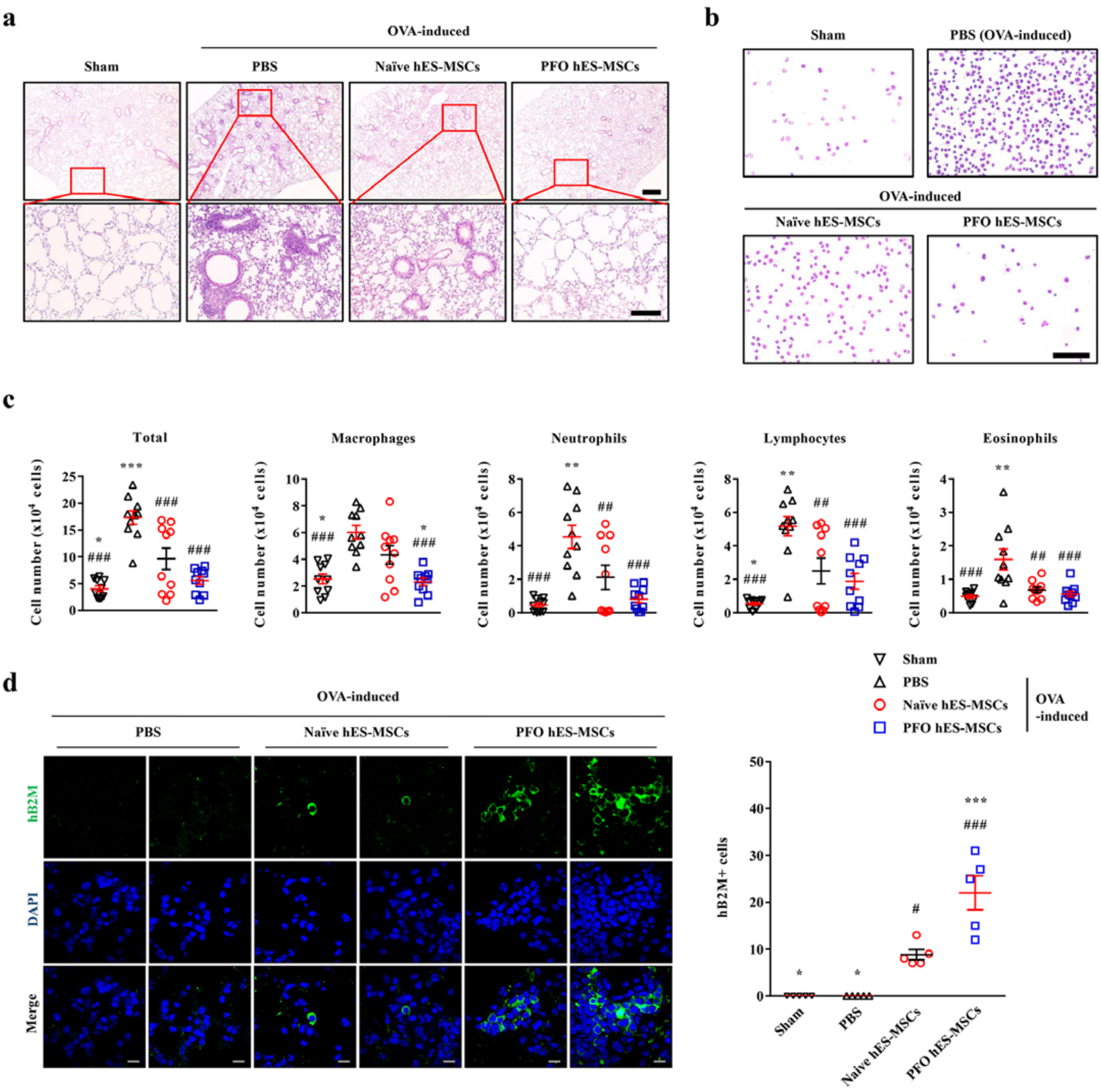

Supplementary Fig. 11 Improved *in vivo* therapeutic efficacy of PFO hES-MSCs for treating asthma

(a) Hematoxylin and eosin staining of lung tissues (magnification, ×40, scale bar, 250 μm) from sham and OVA-induced mice injected with the vehicle control (PBS) or hES-MSCs in normal (Naïve) culture or subjected to the PFO procedure. Higher magnification images (×200)

are shown in the lower panels. (Scale bar, 100 µm.) **(b and c)** Representative images of the cytospin staining (magnification, ×400; scale bar, 50 µm) and the quantification of the numbers of total cells, macrophages, neutrophils, lymphocytes, and eosinophils in the BALF from mice (n = 10) in the indicated groups. **(d)** Immunostaining to detect the engrafted cells expressing hB2M (green) in the lung tissues of OVA-stimulated asthmatic mice one week after injection of the PBS vehicle or the naïve or PFO hES-MSCs (magnification, ×1000; scale bar, 200 µm). The engrafted hB2M^{+} cells in lung tissues from mice (n = 5) in the indicated groups were quantified and are shown in the right panel. All the quantitative data are shown as the mean ± *SEM*. Statistical significance was examined via a one-way ANOVA with Bonferroni *post hoc* tests (*p < 0.05, **p < 0.01, ***p < 0.001 compared with the naïve hES-MSC group; #p < 0.05, ##p < 0.01, ###p < 0.001 compared with the PBS group).

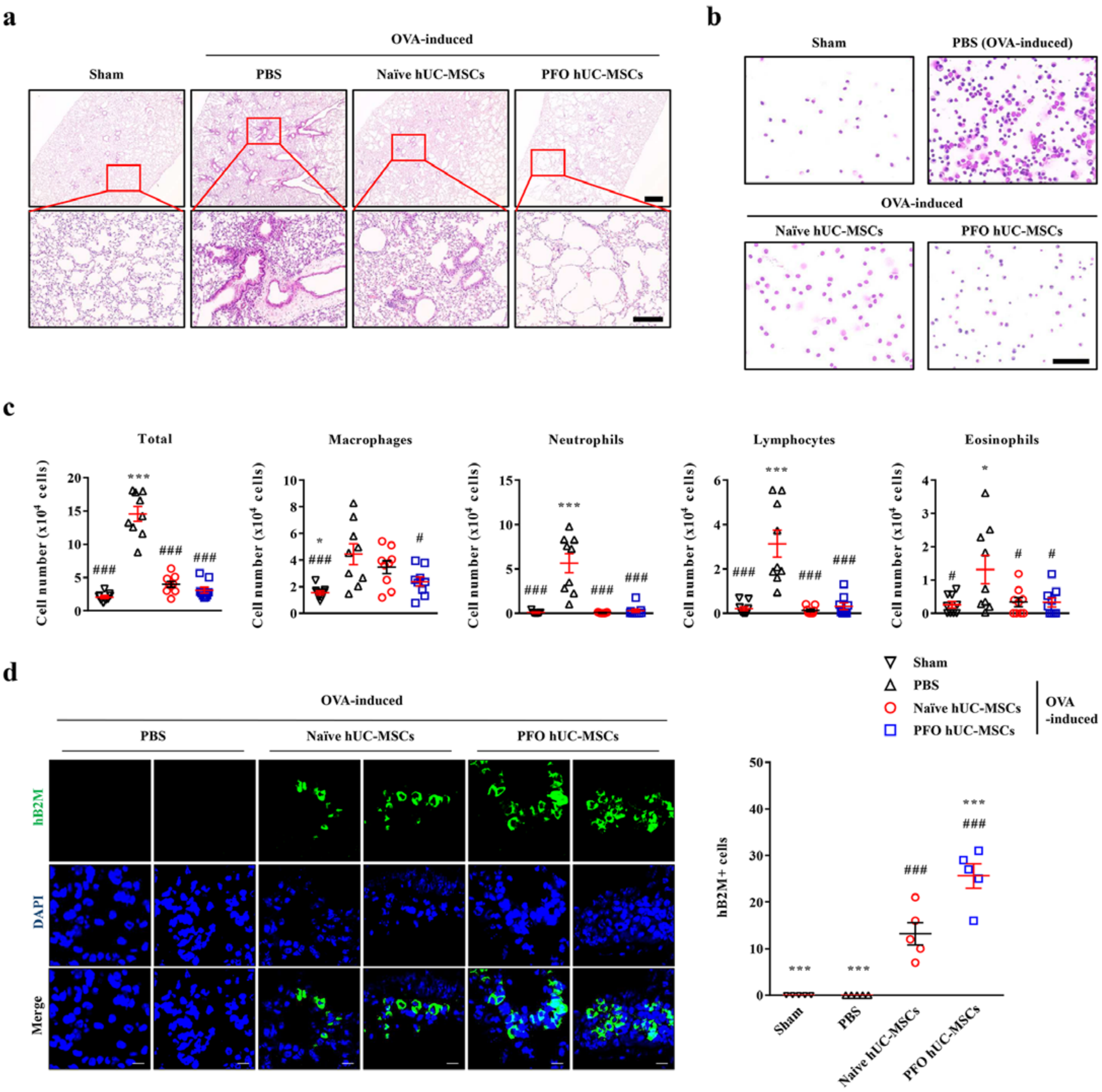

Supplementary Fig. 12 The PFO procedure improved *in vivo* therapeutic potency of hUC-MSCs in a mouse model of allergic asthma

(a) Hematoxylin and eosin staining of lung tissues (magnification, ×40, scale bar, 250 μm) from sham mice and OVA-induced mice injected with the vehicle control (PBS) or hUC-MSCs

in normal (Naïve) culture or subjected to the PFO procedure. Higher magnification images (×200) are shown in the lower panels. Scale bar, 100 µm. **(b and c)** Representative images of the cytospin staining (magnification, ×400; scale bar, 50 µm) and the quantification of the numbers of total cells, macrophages, neutrophils, lymphocytes, and eosinophils in the BALF from mice (n = 9) in the indicated groups. **(d)** Immunostaining to detect the engrafted cells expressing hB2M (green) in the lung tissues of OVA-stimulated asthmatic mice one week after injection of the PBS vehicle or the naïve or PFO hUC-MSCs (magnification, ×1000; scale bar, 200 µm). The engrafted hB2M^{+} cells in lung tissues from mice (n = 5) in the indicated groups were quantified and are shown in the right panel. All the quantitative data are shown as the mean ± *SEM*. Statistical significance was examined via a one-way ANOVA with Bonferroni *post hoc* tests (*p < 0.05, ***p < 0.001 compared with the naïve hUC-MSC group; #p < 0.05, ###p < 0.001 compared with the PBS group).

SUPPLEMENTARY TABLES

Supplementary Table 1. Sequences of the shRNA oligonucleotides

Oligonucleotide (shRNA)	Target sequence
Human_sh*ATF2*_#1	GAAGAAGTGGGTTTGTTTA
Human_sh*ATF2*_#2	GTTGGCGAGTCCATTTGAG
Human_sh*ATF2*_#3	GCATCATTACAGGTTCCCAAT

Supplementary Table 2. Primers used in RQ-PCR analyses

Oligonucleotide (RQ-PCR)	Sense primer	Antisense primer
Human *ATF2*	AAGGTCATGGTAGCGGATTGG	AGTGGATGTGGCTGGCTGTT
Human *CCNA1*	GGATGGCATTTGAGGATGTGTAT	TGTGTTGAAATCCAGCAGGAAGT
Human *CCND1*	CCACCACGGCGTTGTACCT	CATACCCAAAACTTGAACAAATTCC
Human *CRIP2*	GCAAGAAGGTGTACTTCGCTGAG	GCAGGGCTTGTGGCAGTAG
Human *GCLC*	GGAGGAAACCAAGCGCCAT	CTTGACGGCGTGGTAGATGT
Human *GCLM*	TGTCTTGGAATGCACTGTATCTC	CCCAGTAAGGCTGTAAATGCTC
Human *GSR*	TTCCAGAATACCAACGTCAAAGG	GTTTTCGGCCAGCAGCTATTG
Human *HSPA5*	CGCATCACGCCGTCCTAT	TTGGAGGTGAGCTGGTTCTTG
Human *MMP2*	CTGCGGTTTTCTCGAATCCA	GGGTATCCATCGCCATGCT
Human *NQO1*	GGCTAGGTATCATTCAACTCTCCAA	CTTCTCTGAGCAATTCCCTTCTG
Human *NRF2*	TGAGGATTCCTTCAGCAGCAT	GACTGTGGCATCTGAATTTAATGAGT
Human *PDGFRA*	CTTTTTGTGACGGTCTTGGAAGT	TGTCTGAGTGTGGTTGTAATAGCAAG
Human *PLAU*	ATTACTGCAGGAACCCAGACAAC	GCACTCTTGGACAAGCAGCTT
Human PRDX1	CATTCCTTTGGTATCAGACCCG	CCCTGAACGAGATGCCTTCAT
Human *RB1*	CCTTGCATGGCTCTCAGATTC	CAAGCAGATTCAAGGTGATCAGTT

SUPPLEMENTARY REFERENCES

1. Jeong, E. M. *et al.* Real-Time Monitoring of Glutathione in Living Cells Reveals that High Glutathione Levels Are Required to Maintain Stem Cell Function. *Stem Cell*

Reports **10**, 600-614 (2018).

2. Lim, J. *et al.* Glutathione dynamics determine the therapeutic efficacy of mesenchymal stem cells for graft-versus-host disease via CREB1-NRF2 pathway. *Sci. Adv.* **6**, eaba1334 (2020).

UNCROPPED WESTERN BLOT RESULTS

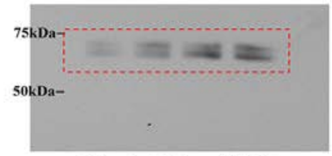

Fig. 1c left t-ATF2

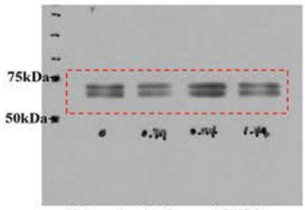

Fig. 1c left p-ATF2

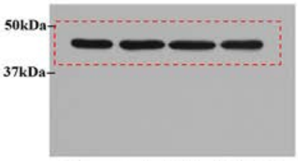

Fig. 1c left β-ACTIN

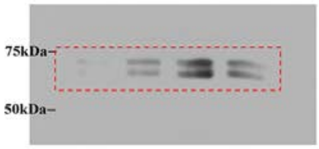

Fig. 1c right t-ATF2

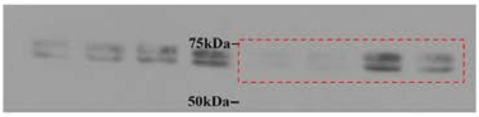

Fig. 1c right p-ATF2

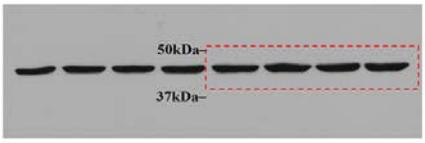

Fig. 1c right β-ACTIN

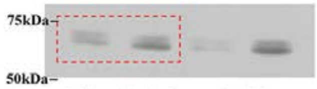

Fig. 1h left t-ATF2

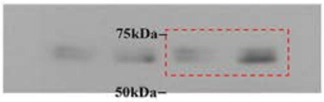

Fig. 1h left p-ATF2

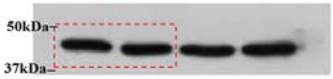

Fig. 1h left β-ACTIN

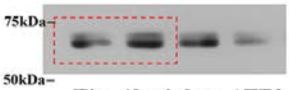

Fig. 1h right t-ATF2

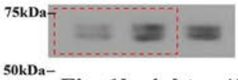

Fig. 1h right p-ATF2

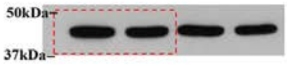

Fig. 1h right β-ACTIN

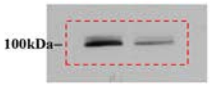

Fig. 2c left NRF2

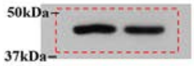

Fig. 2c left β-ACTIN

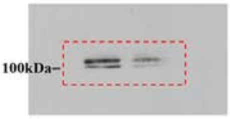

Fig. 2c right NRF2

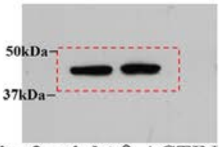

Fig. 2c right β-ACTIN

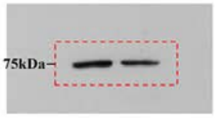

Fig. 2e left GCLC

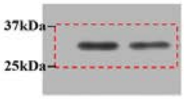

Fig. 2e left GCLM

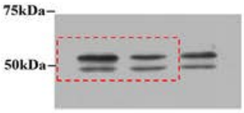

Fig. 2e left GSR

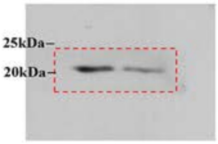

Fig. 2e left PRDX1

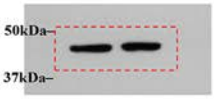

Fig. 2e left β-ACTIN

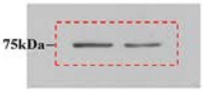

Fig. 2e right GCLC

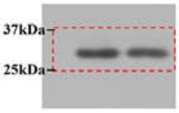

Fig. 2e right GCLM

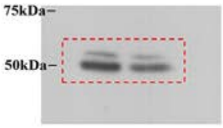

Fig. 2e right GSR

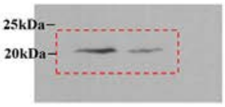

Fig. 2e right PRDX1

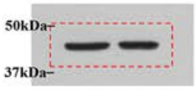

Fig. 2e right β-ACTIN

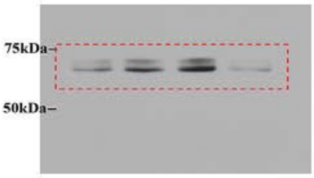

Supple Fig. 1d t-ATF2

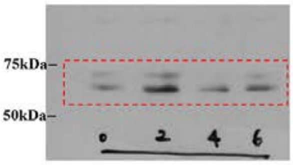

Supple Fig. 1d p-ATF2

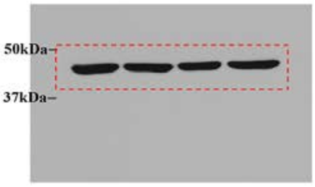

Supple Fig. 1d β-ACTIN

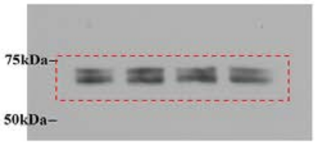

Supple Fig. 1f t-ATF2

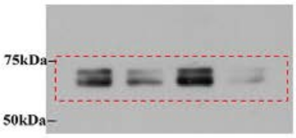

Supple Fig. 1f p-ATF2

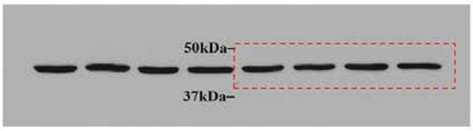

Supple Fig. 1f β-ACTIN

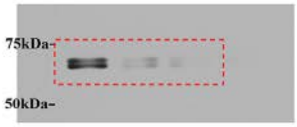

Supple Fig. 2a t-ATF2

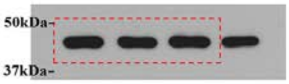

Supple Fig. 2a β-ACTIN

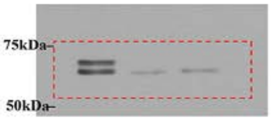

Supple Fig. 2b t-ATF2

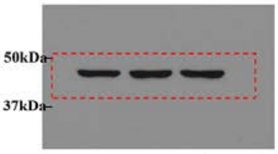

Supple Fig. 2b β-ACTIN

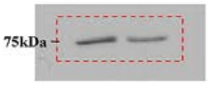

Supple Fig. 2d left
GCLC

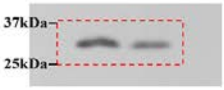

Supple Fig. 2d left
GCLM

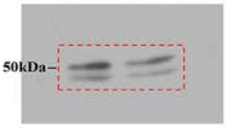

Supple Fig. 2d left
GSR

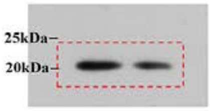

Supple Fig. 2d left
PRDX1

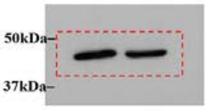

Supple Fig. 2d left
β-ACTIN

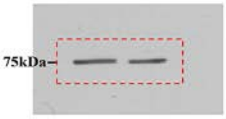

Supple Fig. 2d right
GCLC

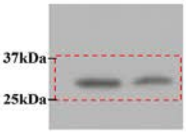

Supple Fig. 2d right
GCLM

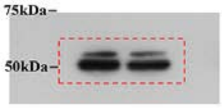

Supple Fig. 2d right
GSR

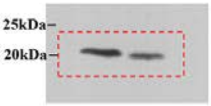

Supple Fig. 2d right
PRDX1

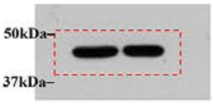

Supple Fig. 2d right
β-ACTIN

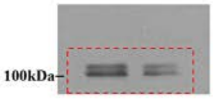

Supple Fig. 2f left
NRF2

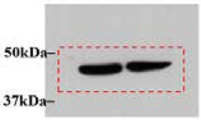

Supple Fig. 2f left
β-ACTIN

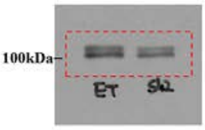

Supple Fig. 2f right
NRF2

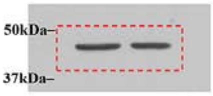

Supple Fig. 2f right
β-ACTIN

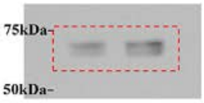

Supple Fig. 7d
t-ATF2

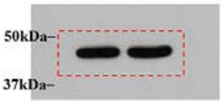

Supple Fig. 7d
β-ACTIN
